# The effects of nine types of exercise rehabilitation therapies on improving limb balance, cognitive and emotional function, and quality of life in elderly patients with Parkinson’s disease: a network meta-analysis of 55 RCTs

**DOI:** 10.3389/fneur.2025.1666552

**Published:** 2025-08-26

**Authors:** Jing Mao, Yi Xia, Yimin Hu, Xuewu Yao

**Affiliations:** ^1^Shanghai University of Sport, Shanghai, China; ^2^Nanchang University, Nanchang, Jiangxi, China

**Keywords:** Parkinson’s disease, exercise therapy, elderly patient, network meta-analysis, balance, cognitive emotions

## Abstract

**Background:**

Parkinson's disease (PD) is a common neurodegenerative disorder that primarily affects individuals over the age of 60. Impaired limb balance, cognitive decline, and emotional disturbances are core symptoms of PD, significantly impacting patients' quality of life. While medication can alleviate motor symptoms, its effectiveness in improving non-motor symptoms (such as cognitive and emotional disturbances) is limited, and long-term use may lead to adverse effects. In recent years, exercise therapy has garnered increasing attention due to its safety, accessibility, and potential to offer both motor and non-motor benefits, making it an important direction in PD rehabilitation research. This study systematically evaluated nine exercise rehabilitation interventions to provide evidence-based non-pharmacological alternatives for PD management.

**Methods:**

A systematic search of six major databases was conducted, and 55 randomized controlled trials involving 4,417 patients with Parkinson's disease were included. The outcome measures were evaluations of balance, cognition, Emotional Functions, and quality of life-related indicators. Stata 17.0 was used to perform a net meta-analysis to assess the relative effectiveness of each intervention and to test the consistency of direct and indirect evidence.

**Results:**

Exoskeletal Training (ET) was the most effective intervention for improving balance (SMD = −2.52, 95% CI [−3.38, −1.67], *p* < 0.0001), resistance training (RT) provided the greatest benefit for reducing Emotional Functions (SMD = 1.02, 95% CI [0.67, 1.38], *p* < 0.0001). In terms of enhancing cognitive function, mind-body exercise (MBE) emerged as the optimal choice (SMD = −1.42, 95% CI [−2.01, −0.84], *p* < 0.0001), while resistance training (RT) was most effective in improving quality of life (SMD = 1.83, 95% CI [0.41, 4.07], *p* < 0.0001).

**Conclusion:**

Dance (DA) is the most effective intervention for improving balance, while aquatic training (ABT) and resistance training (RT) are most effective for emotional regulation. Mind-body exercise (MBE) demonstrates exceptional efficacy in cognitive function, while resistance training has the greatest impact on improving quality of life. These findings provide evidence-based guidance for optimizing exercise-based rehabilitation for Parkinson's disease, supporting tailored interventions targeting specific symptom domains. Future research should focus on refining protocols to maximize treatment efficacy.

## 1 Introduction

Parkinson’s disease (PD) is a neurodegenerative disorder characterized by the progressive degeneration of dopaminergic neurons in the substantia nigra (1). Its core clinical symptoms include resting tremor, muscle rigidity, bradykinesia, and postural instability, often accompanied by non-motor symptoms such as cognitive impairment, depression, anxiety, and sleep disorders (2). According to data from the Global Burden of Disease Study, the global PD patient population has exceeded 6 million, with incidence rates significantly increasing with age, reaching approximately 1–2% in individuals aged 65 and older (3). The pathophysiological basis of the disease primarily involves the selective loss of dopaminergic neurons in the substantia nigra pars compacta (4), leading to a significant decrease in dopamine levels in the striatum and disrupting the functional balance of the basal ganglia motor regulation circuit (5). As the disease progresses, approximately 40% of patients develop Parkinson’s disease dementia (6), and the prevalence of depressive symptoms reaches as high as 40% (7), significantly impairing patients’ quality of life (8). Current clinical interventions primarily focus on slowing disease progression and alleviating symptoms (9).

Mainstream treatment approaches for Parkinson’s disease primarily include pharmacological therapy and surgical intervention (10, 11). However, both have significant limitations. Pharmacological treatment centers on levodopa-based medications (12), which effectively alleviate motor symptoms but may lead to end-of-dose phenomena and dyskinesia upon long-term use (13). Surgical interventions such as deep brain stimulation can significantly improve symptoms in moderate-to-severe patients (14), but they carry surgical risks, are costly, and may result in complications such as infections (15).

Physical therapy, including mind–body exercises, resistance training, and aquatic exercises, can slow neurodegeneration through mechanisms such as regulating brain-derived neurotrophic factor (BDNF) (16). For Parkinson’s patients, exercise therapy has unique advantages (17). However, most studies have focused on patients in the early to mid-stages of the disease, and there is a lack of evidence-based guidelines for elderly PD patients (18); secondly, the optimal combination of different types of exercise (e.g., aerobic vs. resistance training) remains unclear (19); furthermore, the mechanisms by which exercise interventions affect non-motor symptoms (e.g., cognition, depression) in elderly PD patients remain controversial (20). Currently, the optimal exercise regimen and individualized protocols require further evidence-based research, particularly in elderly patients (21). Overall, exercise therapy is a safe, non-pharmacological method that can improve the quality of life of Parkinson’s patients by enhancing motor function and non-motor symptoms such as cognition and mood (22).

This study used a network meta-analysis to compare the effects of nine exercise therapies on balance, mood, and other functional outcomes (23). It analyzed multiple dimensions, including physiological aspects, cognitive levels, and emotional functions, ultimately aiming to improve patients’ overall quality of life. This study provides valuable insights for offering safe and effective non-pharmacological options for Parkinson’s patients, reducing long-term medical burdens, and guiding clinical rehabilitation practices.

## 2 Methods

This study was guided by the Preferred Reporting Items for Systematic Reviews and Meta-Analyses (PRISMA) checklist for network meta-analyses (NMAs10) and the Cochrane Handbook for Systematic Reviews of Interventions. Registration number: CRD420251059397.

### 2.1 Data sources

We conducted a systematic search on PubMed, Embase, Web of Science Cochrane, EBSCO, and China National Knowledge Infrastructure (CNKI1), with two researchers independently selecting studies for inclusion. In PubMed and Cochrane, we used terms from the Medical Subject Headings (MeSH) database for the search. In Embase, we used terms from the Emtree database, and in CNKI, we combined subject terms with free-text keywords for the search. Additionally, we manually screened the reference lists of relevant articles to identify other studies that might meet the inclusion criteria. The search period ranged from January 2000 to June 2025, and only human studies published in Chinese or English were included, with Chinese studies limited to core journals.

The search strategy followed the evidence-based medicine PICOS principles: (P) Population: elderly Parkinson’s disease patients; (I) Intervention: 1. Regular Aerobic Training (RAT); 2. Gait Stability Training (GST); 3. Mind–Body Exercise (MBE); 4. Sensory Stimulation Training (SST); 5. VR Games Training (VRGT); 6. Exoskeletal Training (ET); 7. Resistance Training (RT); 8. Aqua-Based Training (ABT); 9. Dance (DA); (C) Control group: Control group is other exercise therapies; (O) Outcomes: Physical function was assessed using the BA (balance) index, measurement using scales (e.g., Balance Pull Test, BBS, Mini-BES Test, ABC); cognitive function using the COG (cognitive) index, measure using scales (e.g., TMT-B, reaction time, PDCRS total score): emotional regulation using the EF (Emotional Function) index, measurement using scales (e.g., HADS-anxiety, HADS-depression, BDI, GDS-15, PDQ-39, Emotional well-being, SDS); and quality of life using the QOL (quality of life) index, measurement using scales (e.g., Impairment in daily life, PDQ39, PDQ39_Total).(S) Study type: RCTs.

### 2.2 Study selection

Medical search terms were used to conduct searches in PubMed and Cochrane. Additionally, to ensure the comprehensiveness and accuracy of the literature, the reference lists of relevant articles were manually screened to identify other studies that may meet the criteria. It was ensured that literature related to exercise therapy and Parkinson’s disease was retrieved.

After obtaining the initial literature, a rigorous screening process was conducted on these documents. First, EndNote software was used to perform an automatic duplicate literature check to eliminate duplicate records that may have appeared in the database due to differences in search strategies or data sources. Subsequently, duplicate items not automatically identified during the screening process were further removed through manual review of titles and abstracts to ensure the uniqueness and representativeness of the screened literature. For the remaining literature, a more rigorous review was conducted. The following types of studies were primarily excluded: studies targeting non-Parkinson’s patient populations, studies that did not assess relevant indicators, and studies that did not use exercise therapy interventions. Additionally, review articles, conference abstracts, animal studies, research protocols, case reports, retrospective studies, and book chapters were excluded because they typically lack sufficient original data or scientific validity to provide specific analyses and conclusions regarding the effectiveness of interventions. By adopting these stringent literature screening criteria, the final included studies provide high-quality evidence to support this research, further enhancing its scientific validity and credibility.

### 2.3 Eligibility criteria

We included randomized clinical trials in patients with diagnosed Parkinson’s disease (Includes randomized clinical trials for patients with), comparing the effects of different treatments.

Studies meeting the following criteria were eligible for inclusion: (1) were RCTs; (2) involved patients with Parkinson’s disease; (3) Complete outcome measure data; (4) The experimental group received any of the following unconventional movement therapies: RAT, GST, MBE, SST, VRGT, ET, RT, ABT, or DA, while the control group received other movement therapies; (5) At least one of the following measures was assessed: BA (balance), COG (cognitive), EF (Emotional Function), or QOL (quality of life).(6)The age range for the elderly is 60 to 90 years old.

Studies were excluded if they met the following criteria: (1) non-RCTs; (2) animal studies, review articles, conference reports, case reports, letters, or duplicated publications; (3) full-text unavailable; (4) incomplete experimental results or inability to extract data indicators; (5) Failure to report relevant indicators of interest to this study; (6) Patients with other neurological disorders in addition to Parkinson’s disease; (7) Non-core journal articles published in Chinese.

### 2.4 Data collection

Two researchers imported the collected literature into EndNote 20 software according to the search strategy and screened the obtained literature. First, duplicate documents were excluded, followed by a preliminary screening based on titles and abstracts. Full-text articles were then read in detail according to the inclusion and exclusion criteria to further screen the remaining literature. Subsequently, two researchers cross-checked their respective screening results. If they agreed, the literature was included in the study; if there were any discrepancies, a third researcher was consulted, and after discussion and consensus, the literature was finally included.

For eligible trials, two trained researchers independently extracted data from the included literature using standardized data extraction forms and summarized the risk of bias. The extracted data primarily included: (1) basic information of the included literature (first author, publication year, country, etc.); (2) demographic characteristics of the participants (number, age, and gender of the experimental and control groups); (3) detailed information about the intervention (type, intensity, duration, and frequency of the intervention); (4) Outcome measures (mean and standard deviation; primary outcome measures selected included those assessing physical function, cognitive function, emotional regulation, and quality of life; secondary outcome measures selected were used to assess risk of bias). Selected primary outcome measures include those scoring physical function and those scoring cognitive and emotional aspects; selected secondary outcome measures include those rating quality of life. For studies presenting results graphically without numerical summaries, numerical data were extracted using a validated graph digitization tool (GetData 2.22) for analysis. When necessary, we contacted the article authors to obtain information.

### 2.5 Data risk of bias of the systematic review

Using the Cochrane 5.1 version bias risk assessment tool (which includes seven domains: random sequence generation, allocation concealment, blinding of participants and personnel, blinding of outcome assessors, incomplete outcome data, selective reporting, and other biases), two researchers assessed the risk of bias (ROB) for all eligible studies. Risk assessment analysis was conducted using Review Manager 5.3 (Scandinavian Cochrane, Denmark), with each domain assessed as unclear, low risk, or high risk. Based on these assessments, we classified the overall risk of bias for each study as follows: (1) Low ROB: No domains assessed as high risk, and possibly some assessed as unclear but fewer than three; (2) Moderate ROB: One domain assessed as high risk, but no more than one; or no high-risk domains, but more than three domains assessed as unclear; (3) High ROB: All other cases not falling under the above categories were classified as high risk.

### 2.6 Statistical analysis

In this study, META analyzed the data using STATA 17.0 software (Stata Corp LLC, College Station, TX, United States), with the outcome measures being continuous variables. The NMA integrated the pre- and post-intervention changes in both the experimental and control groups to systematically assess the effects of exercise therapy on balance, cognition, Emotional Functions, and quality of life indicators in Parkinson’s patients. To accurately assess the effectiveness of these interventions, the standardized mean difference (SMD) and its 95% confidence intervals (CI) were calculated for each indicator. The baseline was uniformly adjusted to *α* = 0.05, and effect estimates were synthesized using a random-effects model to account for heterogeneity among studies in terms of participant characteristics and intervention methods. We used the following method to calculate the standard deviation of change scores based on the Cochrane Handbook guidelines: For studies that reported baseline and endpoint SD values but lacked SD values for change scores, we used the following formula: *SD_change = √(SD_pre^2^ + SD_post^2^–2 × r × SD_pre × SD_post). Here, r is the correlation coefficient between baseline and post-treatment scores. When r was not reported, we used the conservative default value recommended by the Cochrane guidelines (r = 0.8) based on similar randomized controlled trials (RCTs) in the analysis. Heterogeneity was quantified using the I^2^ statistic and Cochran Q test. The relationships between different non-invasive methods were visualized using a network diagram, where lines connecting nodes represent direct comparisons between different non-invasive methods. The size of nodes and the thickness of connecting lines are proportional to the number of studies including that comparison, and the diagram visually represents the relative strength of interventions and their position within the network. Additionally, the drawn network contributions further quantify the contribution of each direct comparison to the entire network, aiding in analyzing the impact of each intervention on the entire network. Furthermore, to assess publication bias in the studies, a corrected comparison funnel plot was used to analyze publication bias for the primary outcome measures. Finally, the cumulative ranking curve under the curve (SUCRA) method was used to calculate the probability of an intervention being the best.

## 3 Result

### 3.1 Study selection

**Figure 1:**
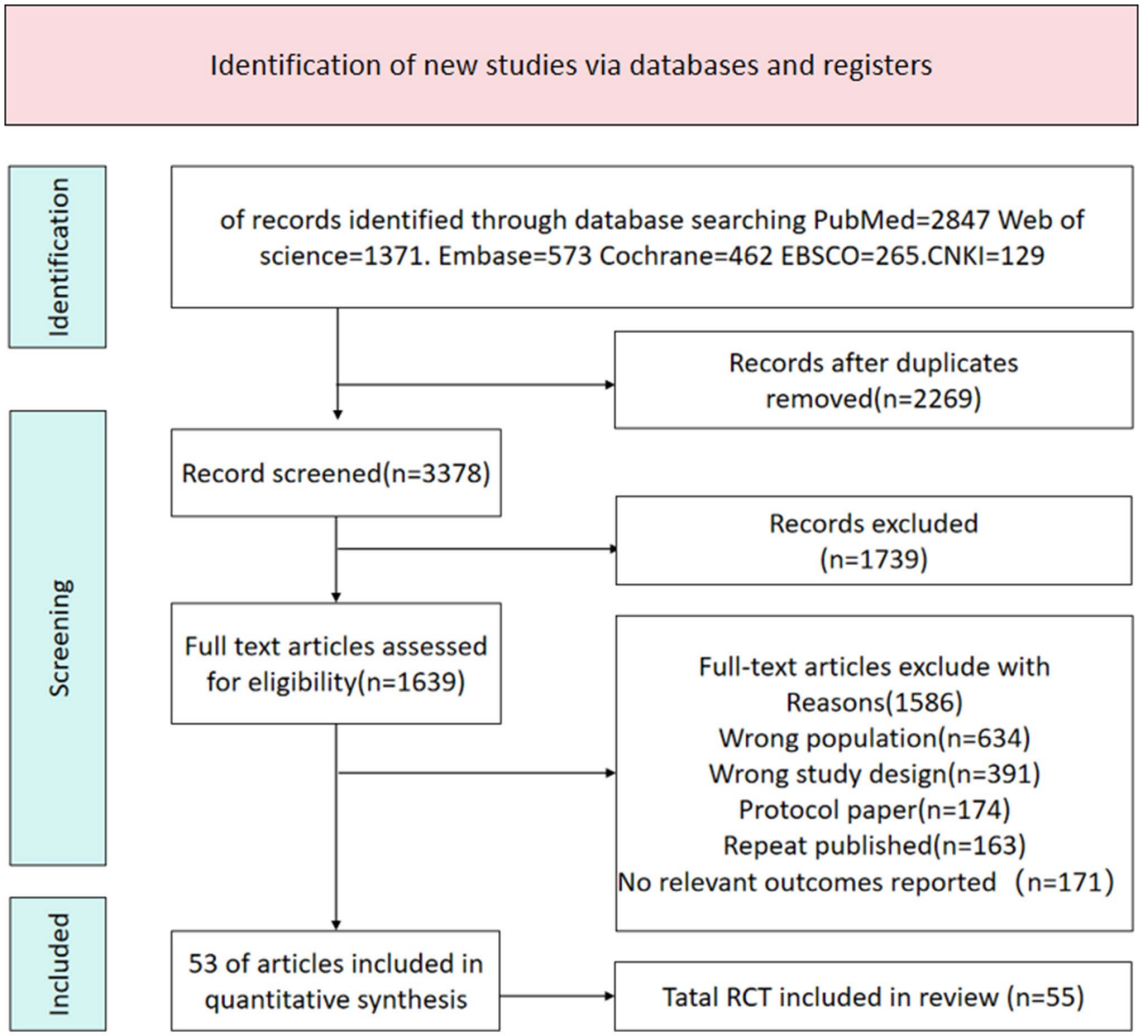
Literature search flowchart.

A total of 5,647 potentially relevant documents were identified through the search. After removing 2,269 duplicate documents, 3,378 articles remained for screening. Through screening of titles and abstracts, 1,739 documents were excluded. According to the inclusion criteria, a further full-text review was conducted, and 1,586 documents that did not meet the criteria were excluded. Ultimately, 53 articles were included. The document selection flowchart is shown in Figure 1.

### 3.2 Literature characterization

A total of 53 articles were included in the analysis, with the basic characteristics of all included studies presented in Table 1. This systematic review and network meta-analysis encompassed studies published between 2007 and 2024, involving multiple countries including Brazil, Italy, China, Canada, Spain, India, Germany, Turkey, Australia, the United States, Japan, Portugal, and Pakistan. The experimental group comprised 2,389 participants who underwent nine types of exercise therapy (RAT, GST, MBE, SST, VRGT, ET, RT, ABT, DA), while the control group included 2,028 participants. In total, 4,417 Parkinson’s disease patients were included, with ages ranging from 60.2 to 77.7 years and an average disease duration of 6.8 years. The duration of exercise intervention ranged from 2 weeks to 12 months, with a median of 8 weeks; each session lasted 20–120 min, with a median of 50 min; sessions were conducted 1–7 times per week, with 3 times being the most common. SST was the most widely used (35.2%), followed by GST (24.1%) and DA control (22.2%). For details, see Table 1.

**Table 1:** Basic features of the included.

Study	Country	Group	Sample size (M/F)	Age (mean ± SD)	Intervention duration	Intervention frequency	认知	PD病程 (年)
Vanbellingen 2017 (45)	Brazil	RT	52 (18/34)	67.15 ± 7.94	4w	30 min, 5 times/week	26.65 ± 1.78	6.12 ± 3.52
		RT	51 (22/29)	68.16 ± 7.38		30 min, 5 times/week	26.59 ± 2.30	6.35 ± 3.99
Agosta 2017 (46)	Italy	SST	13 (8/5)	69.0 ± 8.0	4w	60 min,’3 times/week		
		DA	12 (10/2)	64.0 ± 7.0		60 min, 3 times/week		
Sarasso 2021 (47)	Italy	SST	13 (5/8)	66.41 ± 9.23	6w	60 min, 3 times/week		8.08 ± 4.13
		SST	12 (4/8)	63.86 ± 13.82		60 min, 3 times/week		7.92 ± 3.53
Yang 2016 (48)	China	VRGT	11 (4/7)	72.5 ± 8.4	6w	50 min, twice/week	(MMSE) 27.5 ± 4.0	9.4 ± 3.6
		GST	12 (5/7)	75.4 ± 6.3		50 min, twice/week	27.2 ± 2.5	8.3 ± 4.1
Lin 2024 (49)	China	SST	8 (4/4)	62.63 ± 5.32	8w	25-45 min, twice/week	28.13 ± 0.99	5.38 ± 3.16
		DA	8 (7/1)	63.63 ± 6.48		25-45 min, twice/week	27.50 ± 1.51	5.13 ± 1.55
Nadeau 2014 (50)	Canada	SST	12 (4/8)	64.0 ± 6.6	24w	60 min, 3 times/week	27.8 ± 2.2	
		DA	11 (2/9)	64.3 ± 5.6		60 min, twice/week	(MMSE) 28.6 ± 1.1	
Ferraz 2018 (51)	Brazil	CRT	22 (6/16)	71 ± 6.4	8w	50 min, 3 times/week	27.00 ± 1.1	4 ± 3
		DA	20 (9/11)	67 ± 4.4		50 min, 3 times/week	27.00 ± 1.1	6 ± 4
San 2020 (52)	Spain	SST	23 (12/11)	66.38 ± 7.06	10w	60 min, twice/week		
		GST	17 (5/12)	64.75 ± 8.77		60 min, twice/week		
Alagumoorthi 2022 (53)	India	VRGT	96 (45/51)	69.7 ± 10	12w	30-40 min, 3 times/week		5.37 ± 3.57
		GST	96 (33/63)	68.5 ± 9.8		30-40 min, 3 times/week		5.34 ± 3
Morris 2009 (54)	Australia	SST	14	68 ± 9.2	2w	45 min, 7 times/week		
		DA	14	66 ± 8.6		45 min, 7 times/week		
Spina 2021 (55)	Italy	ET	11 (5/6)	68.0 ± 6.9	4w	45 min, 5 times/week		6.0 ± 1.7
		GST	11 (4/7)	67.3 ± 4.85		45 min, 5 times/week		5.0 ± 2.3
Capecci 2019 (56)	Italy	ET	48 (29/19)	68.1 ± 9.8	4w	45 min, 5 times/week	MMSE 26.1 ± 2.0	8.9 ± 5.3
		GST	48 (24/24)	67.0 ± 7.6		45 min, 5 times/week	26.8 ± 2.1	8.9 ± 4.3
Poier 2019 (57)	Germany	ART	14 (5/9)	68.50 ± 8.07	10w	60 min, once/week		
		MBE	15 (12/3)	68.87 ± 10.96		60 min, once/week		
Zhu 2020 (58)	China	MBE	19 (7/12)	68.53 ± 1.90	12w	Tai Chi: 40-50 min, 3 times/week regular exercise:40-50 min, twice/week		4.68 ± 0.43
		DA	22 (9/13)	67.77 ± 1.72		40-50 min, 5 times/week		4.00 ± 0.39
Kurt 2018 (59)	Turkey	ABT	20 (9/11)	62.41 ± 6.76	5w	60 min, 5 times/week		
		DA	20 (7/13)	63.61 ± 7.18		60 min, 5 times/week		
Volpe 2014 (60)	Italy	ABT	17	66 ± 8	2 m	60 min, 5 times/week		7.6 ± 4.63
		DA	17	68 ± 7		60 min, 5 times/week		7.5 ± 5.1
Gandolfi 2017 (61)	Italy	VRGT	38 (15/23)	67.45 ± 7.18	7w	50 min, 3 times/week	MMSE 26.77 ± 1.48	6.16 ± 3.81
		GST	38 (10/28)	69.84 ± 9.41		50 min, 3 times/week	28.64 ± 6.96	7.47 ± 3.90
Kwok 2019 (30)	China	MBE	71 (34/37)	63.7 ± 8.2	8w	90 min, once/week		
		RT	67 (39/28)	63.5 ± 9.3		90 min, once/week		
Pompeu 2012 (62)	Brazil	SST	12	67.4 ± 8.1	7w	60 min, twice/week		
		GST	12	67.4 ± 8.1		60 min, twice/week		
Zhou 2024 (63)	China	SST	14 (6/8)	62.29 ± 5.37	8w	90 min, twice/week	(MMSE) 29.00 ± 1.57	6.41 ± 5.14
		DA	14 (5/9)	63.14 ± 9.39		90 min, twice/week	28.86 ± 1.79	4.09 ± 3.92
Wong 2024 (64)	China	GST	11 (5/6)	65.86 ± 5.60	6w	40 min, twice/week	(MMSE) 29 ± 1.1	5.0 ± 1.6
		SST	11 (4/7)	67.63 ± 6.92		40 min, twice/week	28 ± 1	3.0 ± 1.0
Silva 2021 (65)	Brazil	SST	5	64 ± 11.9	8w	twice/week		4.8 ± 1.9
		DA	5	63 ± 12.7		twice/week		3.4 ± 2.9
Fernandes 2015 (66)	Portugal	SST	8 (3/5)	63.4 ± 9.5	6w	60 min, twice/week		7.7 ± 7.5
		GST	7 (1/6)	62.3 ± 12.9				8.8 ± 4.3
Shen 2012 (67)	China	SST	14 (5/9)	63.0 ± 8.5	4w	45 min, 3 times/week		7.1 ± 3.2
		RT	14 (7/7)	66.5 ± 8.6		60 min, 3 times/week		5.8 ± 2.2
Bekkers 2020 (68)	Belgium, Israel, UK, Italy, Netherlands	VRGT	62 (25/37)	71.06 ± 6.3	6w	45 min, 3 times/week	MMSE 27.76 ± 1.7	9.05 ± 5.5
		DA	59 (22/37)	70.86 ± 6.0		45 min, 3 times/week	28.34 ± 1.5	9.55 ± 7.2
Chang 2024 (49)	China	MBE	16 (9/7)	66.31 ± 6.54	12w	60 min, 3 times/week	26.75 ± 3.53	6.75 ± 5.49
		DA	14 (9/5)	64.43 ± 7.37		30 min, 3 times/week	27.36 ± 3.07	6.57 ± 7.92
Li 2024 (69)	China	MBE	32 (15/17)	62.7 ± 5.51	12 m	60 min, twice/week	MMSE 28.80 ± 1.19	13.60 ± 2.71
		DA	31 (9/22)	61.5 ± 5.53		60 min, twice/week	29.00 ± 1.27	12.9 ± 2.51
Cruz 2018 (70)	Spain	ABT	14 (9/5)	65.87 ± 7.090	11w	45 min, twice/week		
		DA	15 (8/7)	66.44 ± 5.726		45 min, twice/week		
Hashimoto 2015 (93)	Japan	ART	15 (12/3)	67.9 ± 7.0	12w	60 min, once/week	MMSE 28.2 ± 2.0	6.3 ± 4.6
		DA	17 (15/2)	62.7 ± 14.9		60 min, once/week	28.5 ± 2.0	7.8 ± 6.2
Bezerra 2022 (71)	Brazil	SST	21	64.6 ± 9.3	4w	60 min, 3 times/week		21.0 ± 2.5
		GST	18	60.7 ± 6.8		60 min, 3 times/week		23.0 ± 2.5
Pelosin 2018 (68)	Italy	SST	32 (18/14)	70.4 ± 4.5	5w	45 min, twice/week	(MMSE) 27.3 ± 2.1	10.7 ± 3.9
		DA	32 (17/15)	72.8 ± 3.1		45 min, twice/week	28.2 ± 1.7	9.5 ± 4.2
Kashif 2024 (72)	Pakistan	VRGT	20 (8/12)	63.20 ± 4.85	12w	60 min, 3 times/week	(MMSE) 26.50 ± 0.68	6.65 ± 1.59
		SST	20 (10/10)	64.85 ± 5.10		everyday	26.40 ± 1.14	6.40 ± 2.50
Landers 2016 (73)	USA	SST	10 (6/4)	72.2 ± 4.4	4w	45 min, 3 times/week	(MMSE) 27.6 ± 1.1	
		GST	10 (7/3)	70.1 ± 9.5		45 min, 3 times/week	29.6 ± 0.5	
Wong-Yu 2015 (74)	China	GST	32 (13/19)	60.2 ± 9.0	8w	120 min, once/week		7.3 ± 4.6
		RT	36 (16/20)	61.9 ± 8.5		120 min, once/week		5.4 ± 3.6
Capato 2020 (75)	Brazil	SST	56 (27/29)	74 ± 8	5w	45 min, twice/week	26 ± 3	5 ± 3
		CNT	48 (19/29)	73 ± 10			24 ± 3	8 ± 3
Steib 2017 (76)	Germany	GST	18 (7/11)	67.6 ± 8.2	8w	40 min, twice/week		7.9 ± 4.0
		DA	19 (4/15)	62.5 ± 7.9		40 min, twice/week		7.3 ± 4.4
Gaßner 2019 (77)	Germany	GST	18 (7/11)	67.6 ± 8.2	8w	30 min, twice/week	25.8 ± 3.7	7.9 ± 4.0
		DA	20 (4/16)	62.5 ± 7.9		30 min, twice/week	25.6 ± 3.9	7.3 ± 4.4
Cheng 2016 (63)	China	GST	12 (4/8)	66.4 ± 7.8	4-6w	40 min, 3 times/week	(MMSE) 28.1 ± 1.8	6.5 ± 2.4
		DA	12 (3/9)	65.8 ± 11.5		40 min, 3 times/week	27.7 ± 1.3	6.1 ± 4.1
Cheng 2016 (63)	Brazil	DA	5 (1/4)	64.8 ± 11.9	12w	twice/week	(MMSE) 24.6 ± 4.0	6.6 ± 1.5
		RT	8 (2/6)	64.1 ± 9.9		twice/week	25.8 ± 3.2	6.0 ± 2.6
Calabrò 2019 (78)	Italy	SST	25 (9/11)	70 ± 8	8w	30 min, 5 times/week	MMSE 26 ± 3	10 ± 3
		DA	25 (6/14)	73 ± 8		30 min, 5 times/week	25 ± 3	9.3 ± 3
Gandolfi 2017 (61)	Italy	VRGT	38 (15/23)	67.45 ± 7.18	7w	50 min, 3 times/week	MMSE 26.77 ± 1.48	6.16 ± 3.81
		GST	38 (10/28)	69.84 ± 9.41		50 min, 3 times/week	28.64 ± 6.96	7.47 ± 3.90
Kim 2022 (79)	South Korea	ET	22 (16/6)	68.7 ± 6.9	4w	45 min, 3 times/week	MMSE 28.4 ± 1.3	9 ± 2
		DA	22 (15/7)	67.5 ± 9.3		45 min, 3 times/week	28.1 ± 1.8	104.6 ± 53.4
Picelli 2015 (80)	Italy	ET	33 (11/22)	68.2 ± 9.2	4w	45 min, 3 times/week		7.5 ± 5.6
		GST	33 (7/26)	69.7 ± 7.2		45 min, 3 times/week		8.3 ± 4.1
Picelli 2013 (81)	Italy	ET	20 (11/9)	68.50 ± 10.10	4w	45 min, 3 times/week		6.52 ± 5.30
		DA	20 (14/6)	68.80 ± 7.72		45 min, 3 times/week		6.99 ± 6.17
Picelli 2012 (82)	Italy	ET	17	68.3 ± 7.5	4w	40 min, 3 times/week		
		CRT	17	68.3 ± 7.5		40 min, 3 times/week		
Li 2022 (83)	China	MBE	32 (15/17)	62.7 ± 5.51	12 m	60 min, 3 times/week		5.91 ± 4.01
		DA	31 (9/22)	61.9 ± 5.64		60 min, 3 times/week		3.82 ± 1.87
Zhang 2015 (84)	China	MBE	20 (7/13)	66.00 ± 11.80	12w	60 min, twice/week	27.40 ± 2.33	6.80 ± 5.43
		DA	20 (9/11)	64.35 ± 10.53		60 min, twice/week	26.35 ± 2.39	4.85 ± 3.72
Xiao 2016 (85)	China	MBE	45 (14/31)	68.17 ± 2.27	6 m	45 min, 4 times/week	MMSE 28.08 ± 1.87	5.45 ± 3.61
		DA	44 (13/31)	66.52 ± 2.13		30 min, 7 times/week	27.9 ± 1.49	6.15 ± 2.63
Cruz 2017 (31)	Spain	ABT	15	66.8 ± 5.267	10w	45 min, twice/week		6.2 ± 2.541
		DA	15	67.53 ± 9.89		45 min, twice/week		6.7 ± 3.225
Vivas 2011 (86)	Spain	ABT	6 (3/3)	65.67 3.67	4w	45 min, twice/week	27.83 ± 2.23	4.17 1.60
		DA	6 (2/4)	68.33 6.92		45 min, twice/week	27.5 ± 2.17	7.83 3.92
Hackney 2007 (87)	USA	ART	9 (3/6)	72.6 2.20	13w	60 min, twice/week		6.2 ± 1.5
		RT	10 (4/6)	69.6 2.1		60 min, twice/week		3.3 ± 0.5
Ni 2016 (88)	USA	CNT	10 (6/4)	74.9 ± 8.3		60 min, once/month		5.9 ± 6.2
		MBE						
Cherup 2021 (89)	USA	MBE	15 (5/10)	69.8 ± 7.3	12w	45 min, twice/week		
		GST	18 (7/11)	71.4 ± 12.1		45 min, twice/week		
Furnari 2017 (90)	Italy	ET	19 (8/11)	71.5 ± 11.7	4w	60 min, 6 times/week		
		GST	19 (9/10)	77.7 ± 8.3		60 min, 6 times/week		
Carda 2012 (91)	Italy	ET	15	67.87 ± 7.05	4w	30 min, 3 times/week	MMSE 25.94 ± 2.04	
		GST	15	66.93 ± 5.13		30 min, 3 times/week	25.52 ± 2.19	

### 3.3 Risk of bias assessment

**Figure 2:**
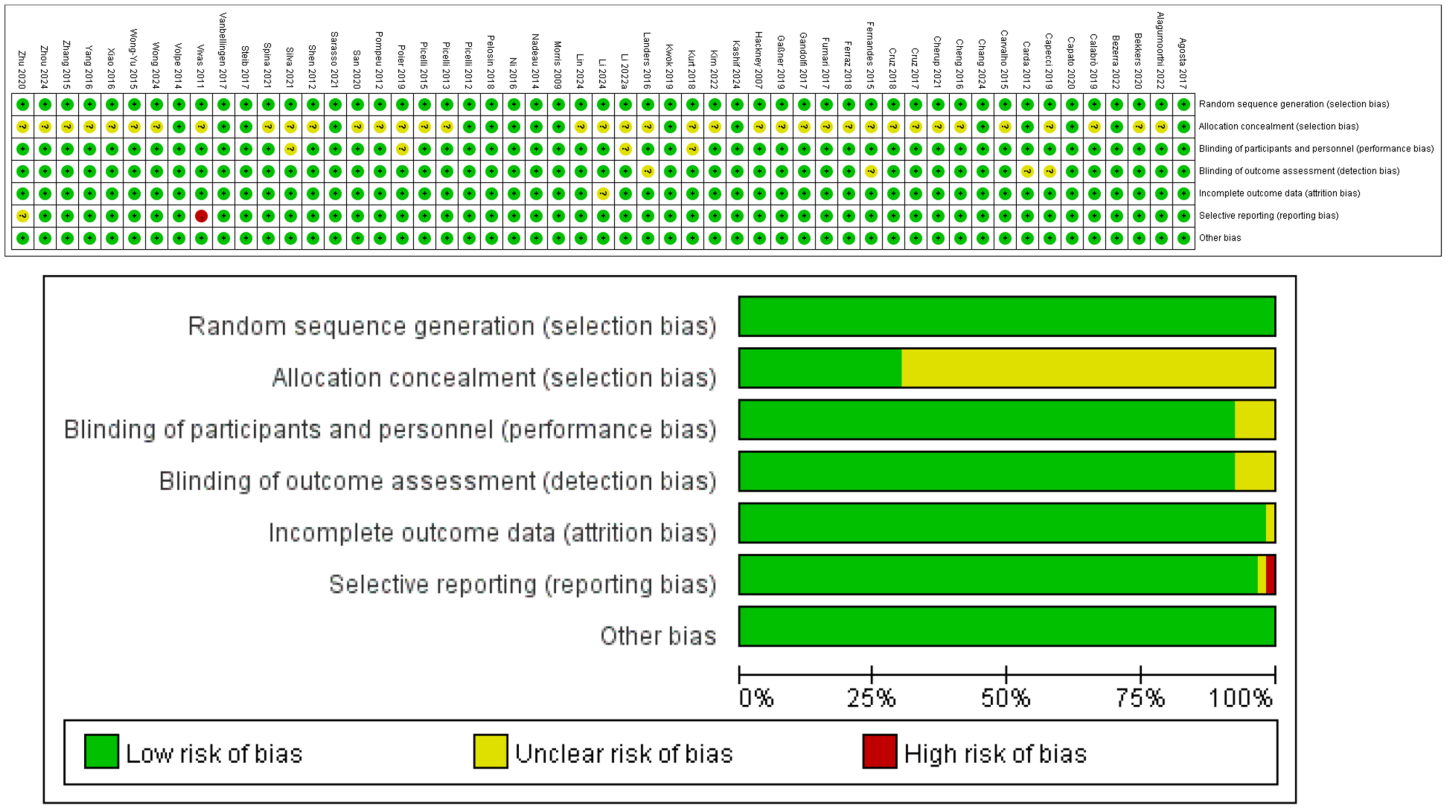
Risk assessment results.

Table 2 and Figure 2 provide detailed information on the ROB assessment for each study. Among the 53 articles, 53 mentioned random allocation, with 16 describing the method of random allocation and 4 describing allocation concealment; 46 reported blinding; 49 reported outcome assessment blinding; and 51 studies showed a low risk of selective reporting; No other biases were identified in any of the articles. In summary,37 articles were judged to have low ROB, 1 article has been judged to have high ROB.

**Table 2:** Evaluation results of literature quality risk bias of included articles.

Inclusion of literature	Random sequence generation	Allocation concealment	Blinding of participants and personnel	Blinding of outcome assessment	Incomplete outcome data	Selective reporting	Other bias
Agosta 2017 (46)	L	L	L	L	L	L	L
Alagumoorthi 2022 (53)	L	U	L	L	L	L	L
Bekkers 2020 (68)	L	U	L	L	L	L	L
Bezerra 2022 (71)	L	L	L	L	L	L	L
Calabrò 2019 (78)	L	U	L	L	L	L	L
Capato 2020 (75)	L	L	L	L	L	L	L
Capecci 2019 (56)	L	U	L	U	L	L	L
Carda 2012 (91)	L	L	L	U	L	L	L
Carvalho 2015 (92)	L	U	L	L	L	L	L
Chang 2024 (49)	L	L	L	L	L	L	L
Cheng 2016 (63)	L	U	L	L	L	L	L
Cherup 2021 (89)	L	U	L	L	L	L	L
Cruz 2017 (31)	L	U	L	L	L	L	L
Cruz 2018 (70)	L	U	U	L	L	L	L
Fernandes 2015 (66)	L	U	L	U	L	L	L
Ferraz 2018 (51)	L	U	L	L	L	L	L
Furnari 2017 (90)	L	U	L	L	L	L	L
Gandolfi 2017 (61)	L	U	L	L	L	L	L
Gaßner 2019 (77)	L	U	L	L	L	L	L
Hackney 2007 (87)	L	U	L	L	L	L	L
Kashif 2024 (72)	L	L	L	L	L	L	L
Kim 2022 (79)	L	U	L	L	L	L	L
Kurt 2018 (59)	L	U	U	L	L	L	L
Kwok 2019 (30)	L	L	L	L	L	L	L
Landers 2016 (73)	L	U	L	U	L	L	L
Li 2022 (83)	L	U	U	L	L	L	L
Li 2024 (69)	L	U	L	L	U	L	L
Lin 2024 (49)	L	U	L	L	L	L	L
Morris 2009 (54)	L	L	L	L	L	L	L
Nadeau 2014 (50)	L	L	L	L	L	L	L
Ni 2016 (88)	L	L	L	L	L	L	L
Pelosin 2018 (68)	L	L	L	L	L	L	L
Picelli 2012 (82)	L	L	L	L	L	L	L
Picelli 2013 (81)	L	U	L	L	L	L	L
Picelli 2015 (80)	L	U	L	L	L	L	L
Poier 2019 (57)	L	U	U	L	L	L	L
Pompeu 2012 (62)	L	U	L	L	L	L	L
San 2020 (52)	L	U	L	L	L	L	L
Sarasso 2021 (47)	L	L	L	L	L	L	L
Shen 2012 (67)	L	U	L	L	L	L	L
Silva 2021 (65)	L	U	U	L	L	L	L
Spina 2021 (55)	L	U	L	L	L	L	L
Steib 2017 (76)	L	L	L	L	L	L	L
Vanbellingen 2017 (45)	L	L	L	L	L	L	L
Vivas 2011 (86)	L	U	L	L	L	H	L
Volpe 2014 (60)	L	L	L	L	L	L	L
Wong 2024 (64)	L	U	L	L	L	L	L
Wong-Yu 2015 (74)	L	U	L	L	L	L	L
Xiao 2016 (85)	L	U	L	L	L	L	L
Yang 2016 (48)	L	U	L	L	L	L	L
Zhang 2015 (84)	L	U	L	L	L	L	L
Zhou 2024 (63)	L	U	L	L	L	L	L
Zhu 2020 (58)	L	U	L	L	L	U	L

### 3.4 Direct pairwise meta-analyses (primary outcome)

**Figure 3:**
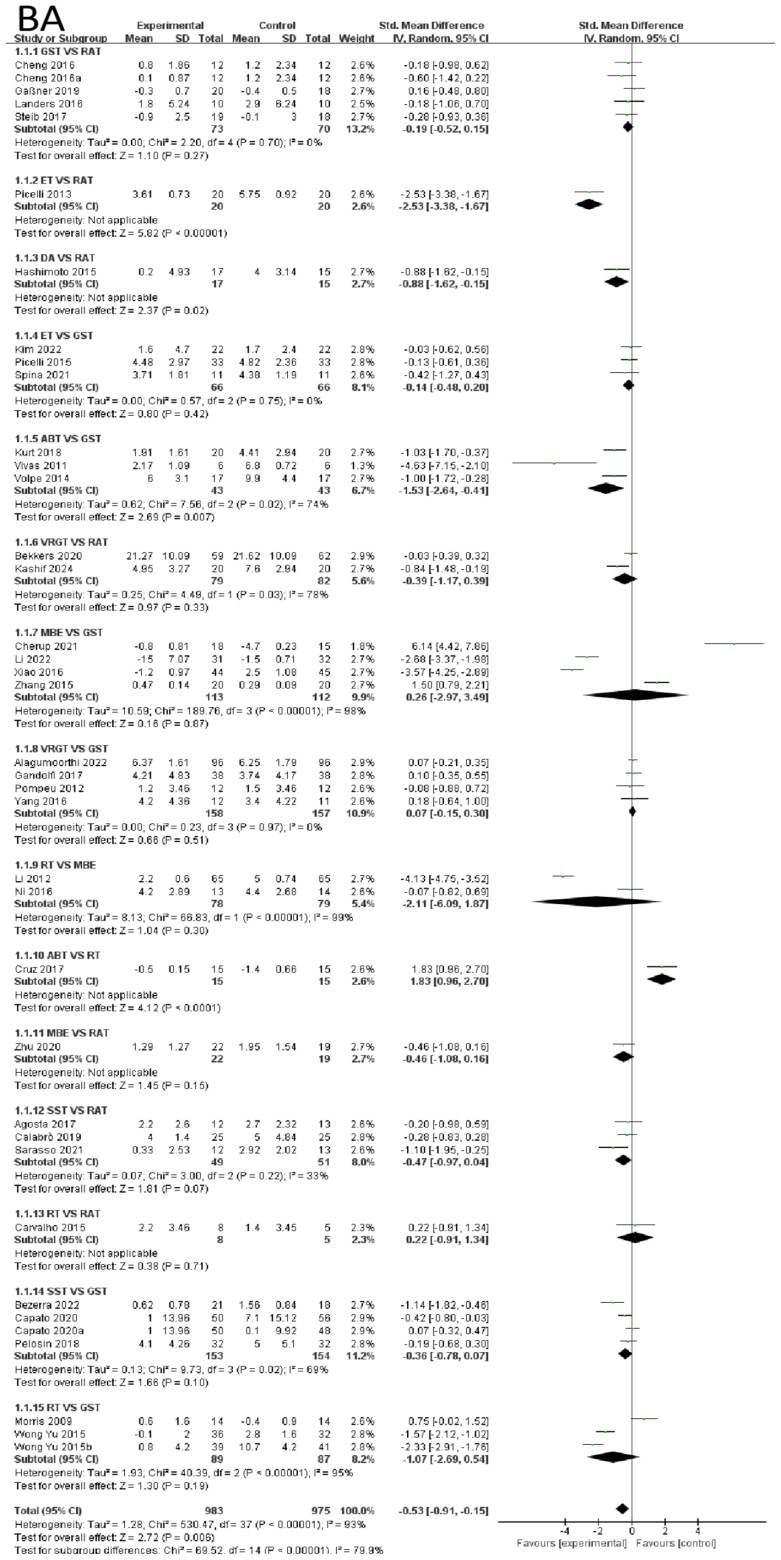
Forest of balance.

First, the effects of various intervention measures on balance outcomes were analyzed among 1,977 participants. The forest plot for balance is shown in Figure 3. The study compared the effects of various intervention measures with the control group (primarily RAT and GST) on improving balance function using forest plot analysis. The results showed that exoskeleton training (ET) demonstrated the most significant therapeutic advantage among all intervention measures. Compared with RAT, the mean difference for ET was −2.53 (95% CI: −3.38, −1.67, *p* < 0.0001), with the largest effect size and high statistical significance, indicating that ET can significantly improve balance function. In contrast, other interventions such as dance (DA) showed moderate improvements. Compared with RAT, the mean difference for DA was −0.88 (95% CI: −1.52, −0.15, *p* = 0.02), although the effect was significant, its effect size was notably smaller than that of ET. This suggests that the effects of ART may vary significantly across different studies. ART (mean difference −1.03, *p* < 0.01) had some effect, but the effect size was small, and ART exhibited moderate heterogeneity (I^2^ = 74%). GST, VRGT, and MBE showed no significant differences compared to the control group (*p* > 0.05), with MBE exhibiting extremely high heterogeneity (I^2^ = 96%). This may be related to methodological differences or variations in participant characteristics across studies. In summary, ET is the optimal intervention for improving balance function based on current evidence, with clear and stable efficacy when compared to RAT. DA and ART also showed some improvement effects, but the effect sizes were small or there were issues of heterogeneity. VRGT, MBE, and other interventions did not show significant advantages in this analysis. These findings provide important evidence for selecting balance function improvement strategies in clinical practice and also suggest that future studies need to further explore the sources of effect differences in interventions with high heterogeneity. See Figure 3 for details.

**Figure 4:**
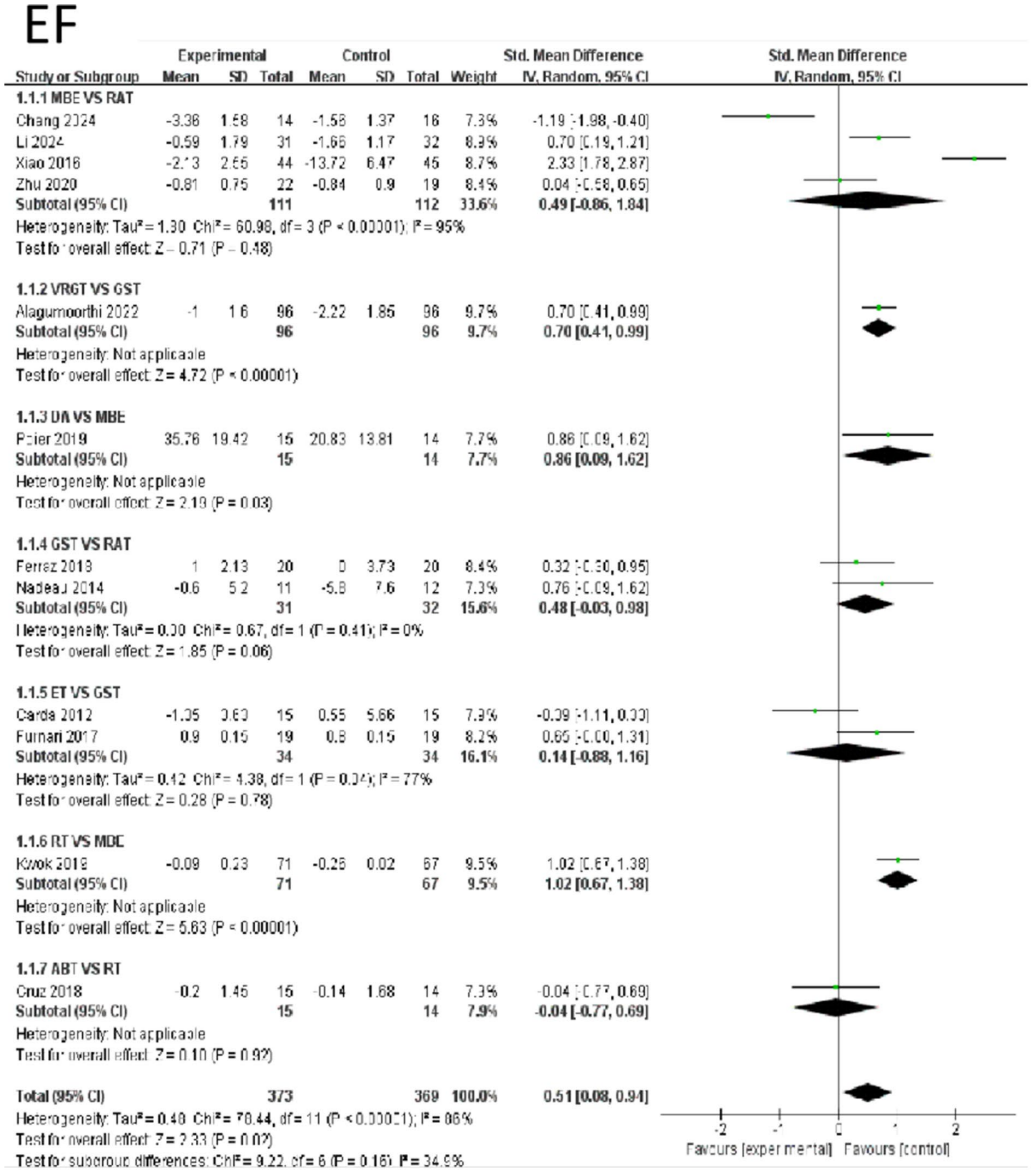
Forest of emotional function.

The effects of various intervention measures on Emotional Function indicators were examined in a study involving 742 participants. The forest plot for Emotional Functions is shown in Figure 4. The results showed that the overall effect size of the study was 0.51 (95% CI: 0.08–0.94), Z = 2.33 (*p* = 0.02), indicating that the interventions had a statistically significant effect on improving Emotional Function overall. However, it is important to note that there was high heterogeneity (I^2^ = 86%, Tau^2^ = 0.48), suggesting significant differences between studies. In subgroup analyses, RT and MBE showed extremely high effect sizes of 1.02 (95% CI: 0.67–1.38), with significant effects (z = 5.63, *p* < 0.0001), indicating that RT has a significant advantage over MBE in improving Emotional Function. Additionally, the comparison effect size between VRGT and GST was 0.70 (95% CI: 0.41–0.99, z = 4.72, *p* < 0.0001), also indicating a significant effect of the intervention group. The effect size for the comparison between GST and RAT was 0.48 (95% CI: −0.03–0.98, Z = 1.85, *p* = 0.06), which was close to significance but did not reach statistical significance. In contrast, other intervention groups such as ABT and MBE also showed some effects, but these were relatively small, and some groups exhibited high heterogeneity. In summary, RT was most effective in improving emotional function, while the effects of other interventions varied. Emotional Function were not associated with other categorical variables (e.g., age, study region, study year). Compared to shorter interventions, longer exercise interventions (8 weeks or longer) were significantly associated with reduced levels of Emotional Function indicators. See Figure 4 for details.

**Figure 5:**
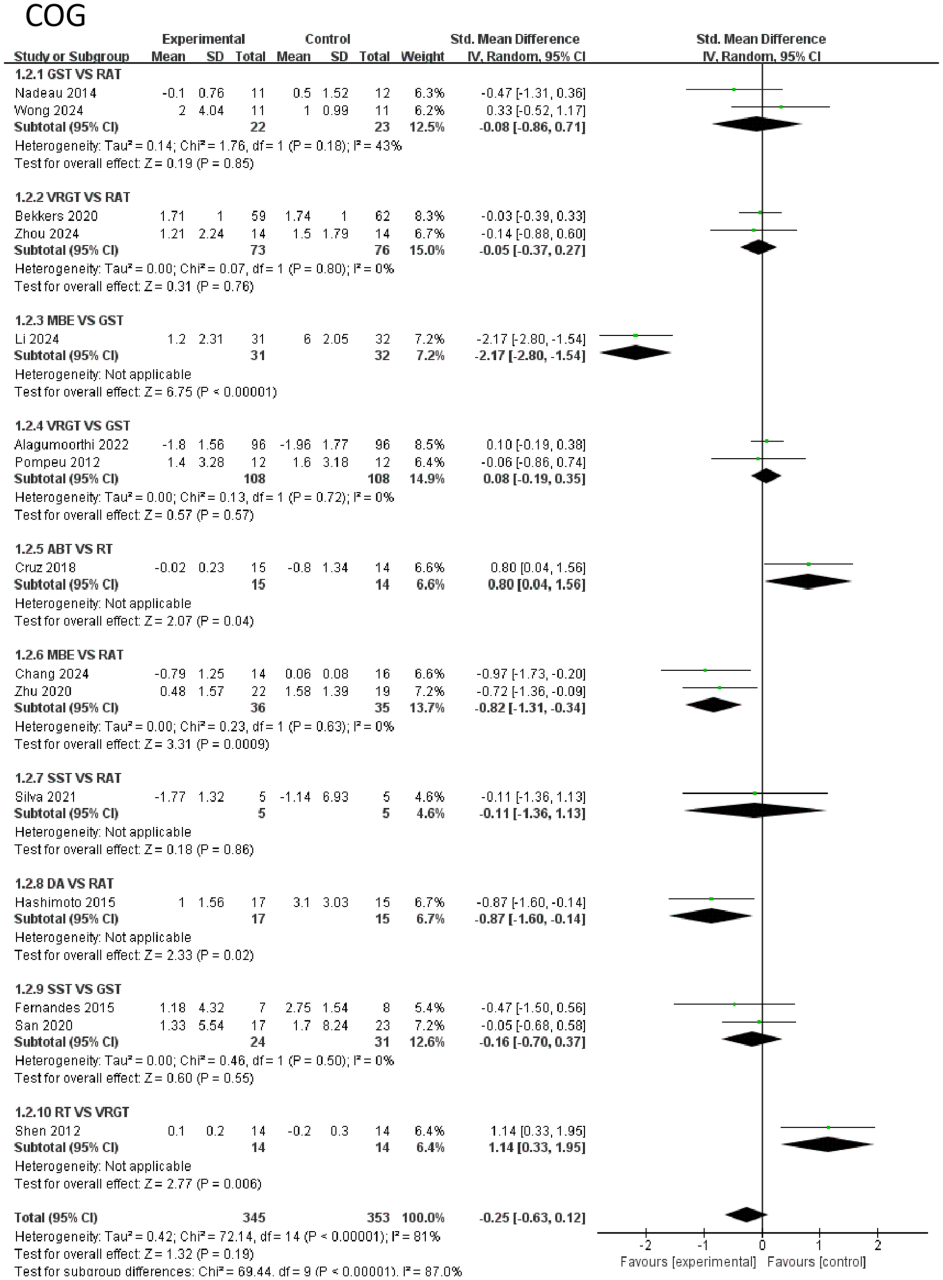
Forest of cognitive.

The effects of 8 intervention measures on cognitive indicators among 698 participants. The overall analysis showed that the pooled standardized mean difference (SMD) for all interventions was not statistically significant (SMD = −0.19, 95% CI [−0.47, 0.09], *p* = 0.19), but subgroup analysis indicated significant heterogeneity in the effects of different interventions (I^2^ = 81%, *p* < 0.0001). Specifically, movement-based interventions (MBE) had the most significant effect on improving cognitive outcomes: the SMD for MBE vs. RAT was −0.79 (95% CI [−1.25, −0.33], *p* = 0.0009), and MBE vs. GST had an SMD of −1.20 (95% CI [−1.57, −0.83], *p* < 0.0001). Additionally, ABT vs. RT (SMD = −0.78, 95% CI [−1.52, −0.04], *p* = 0.04) and DA vs. RAT (SMD = −1.54, 95% CI [−2.82, −0.26], *p* = 0.02) also showed significant advantages, but the sample sizes were small. Differences between other interventions (e.g., VRGT, SST) and the control group were not statistically significant (*p* > 0.05). In summary, MBE is the most effective in improving cognitive indicators. Aerobic exercise and coordination training can increase the secretion of brain-derived neurotrophic factor (BDNF), promote hippocampal neurogenesis and synaptic plasticity, thereby enhancing learning, memory, and executive function. The evidence strength is high (large sample size and extremely low *p*-value), making it a priority intervention strategy. See Figure 5 for details.

**Figure 6:**
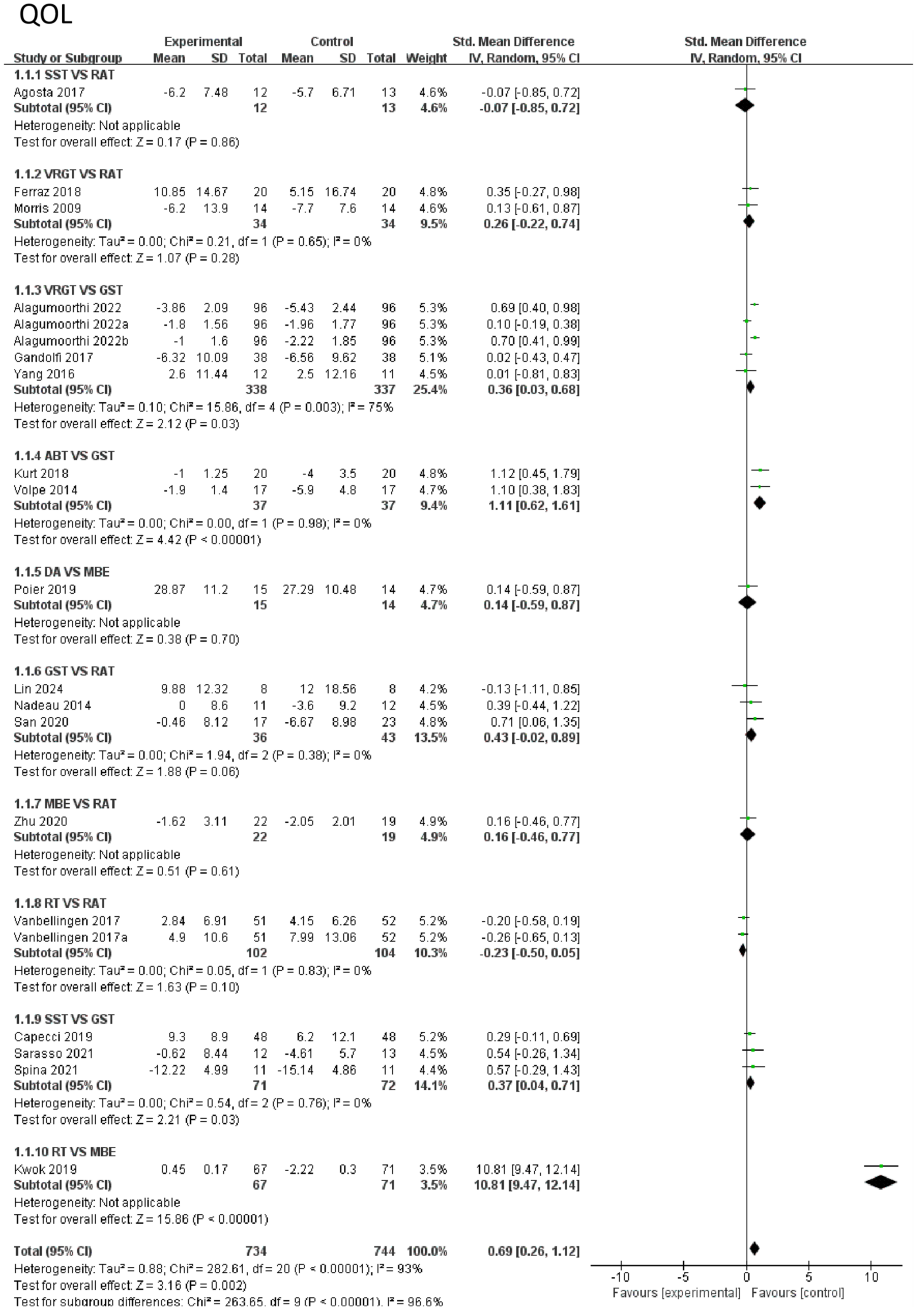
Forest of quality of life.

First, the effects of various intervention measures on quality-of-life indicators were analyzed in a study involving 1,478 participants. The results showed that the overall effect size (mean difference) exhibited high heterogeneity (I^2^ = 93%, *p* < 0.0001). The pooled effect size indicated that exercise therapy significantly improved quality of life (SMD = 0.69, 95% CI [0.26, 1.12], *p* = 0.002), but there were significant differences between different intervention measures (subgroup heterogeneity I^2^ = 96.6%, *p* < 0.0001). Subgroup analysis and regression analysis are detailed in Appendix 2. In subgroup analysis, the comparison between RT and MBE showed the largest effect size (SMD = 10.81, 95% CI [9.47, 12.14], *p* < 0.0001), indicating that RT was significantly superior to MBE. Additionally, ABT demonstrated a significant advantage over GST (SMD = 1.11, 95% CI [0.62, 1.61], *p* < 0.0001). The comparison between VRGT and GST also showed a moderate effect size (SMD = 0.36, 95% CI [0.03, 0.68], *p* = 0.03), while the comparison between SST and GST was also significant (SMD = 0.37, 95% CI [0.04, 0.71], p = 0.03). In contrast, the comparative effects of RAT with other interventions were weaker, such as with GST (SMD = 0.43, 95% CI [−0.02, 0.89], *p* = 0.06) and MBE (SMD = 0.16, 95% CI [−0.46, 0.77], *p* = 0.61). In summary, RT and ABT are the most effective interventions for improving quality of life, followed by VRGT and SST. The effects of RAT and MBE are relatively limited. These results suggest that interventions based on strength (e.g., RT, ABT) and technology-assisted approaches (e.g., VRGT) may be more suitable for enhancing quality of life. See Figure 6 for details.

### 3.5 Network meta-analysis

#### 3.5.1 Network diagram of included studies

**Figure 7:**
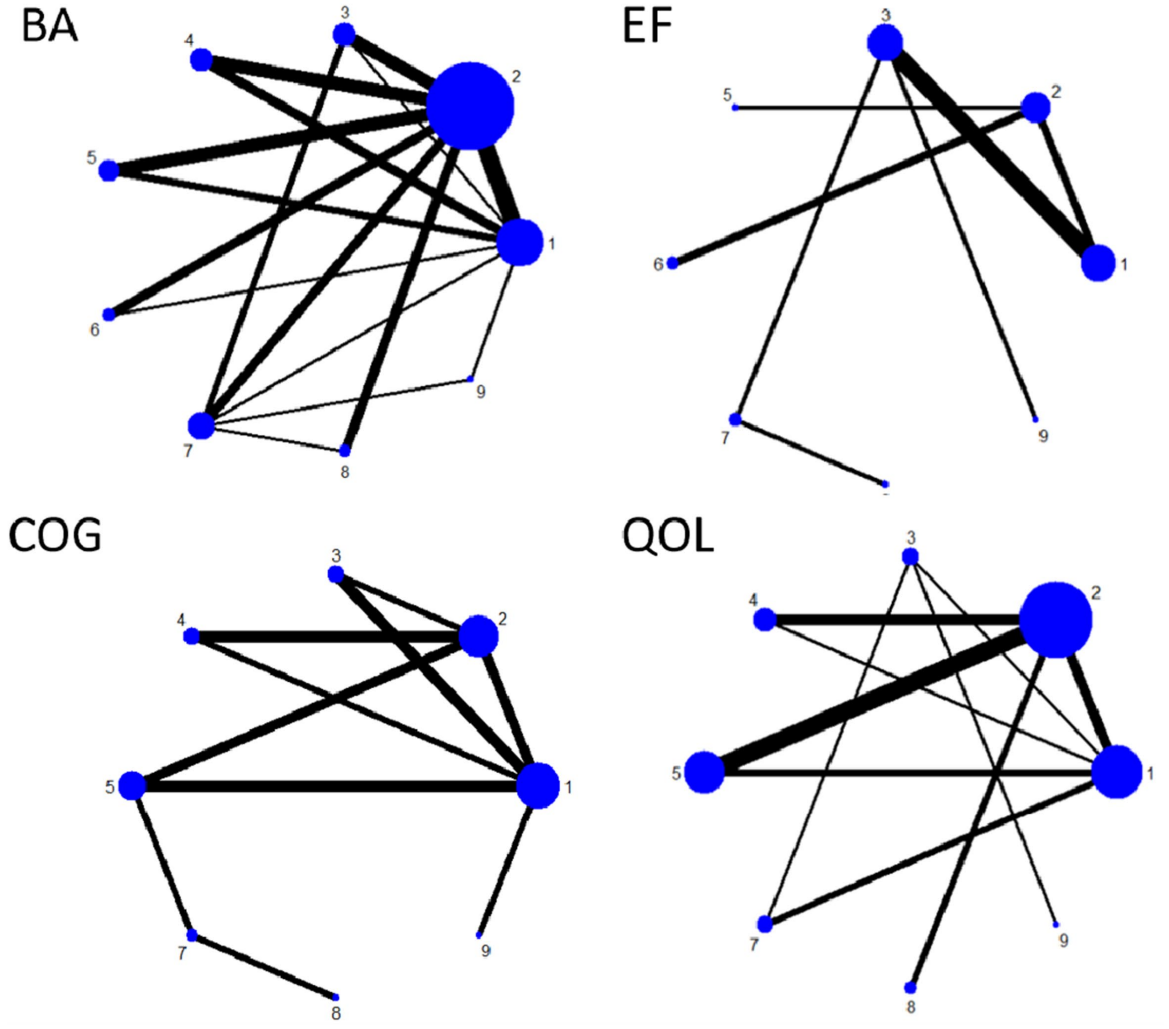
Network plot of outcome indicators.

Figure 7 shows the NMA diagram for nine types of exercise therapy. This diagram evaluates the effectiveness of nine types of exercise therapy. The size of the nodes in the chart reflects the sample size of each type of exercise therapy, and the thickness of the lines between the nodes indicates the number of studies comparing these interventions. GST is the most widely used intervention, while DA and ET have been studied less. The network diagram of the outcome measures is shown in detail in Figure 7. The results of consistency test and local inconsistency test are detailed in Appendix 1.

#### 3.5.2 Ranking of intervention effectiveness of nine exercise therapy

*BA indicator*: Effective ranking of 14 types of exercise therapy on balance in elderly Parkinson’s patients.

**Figure 8:**
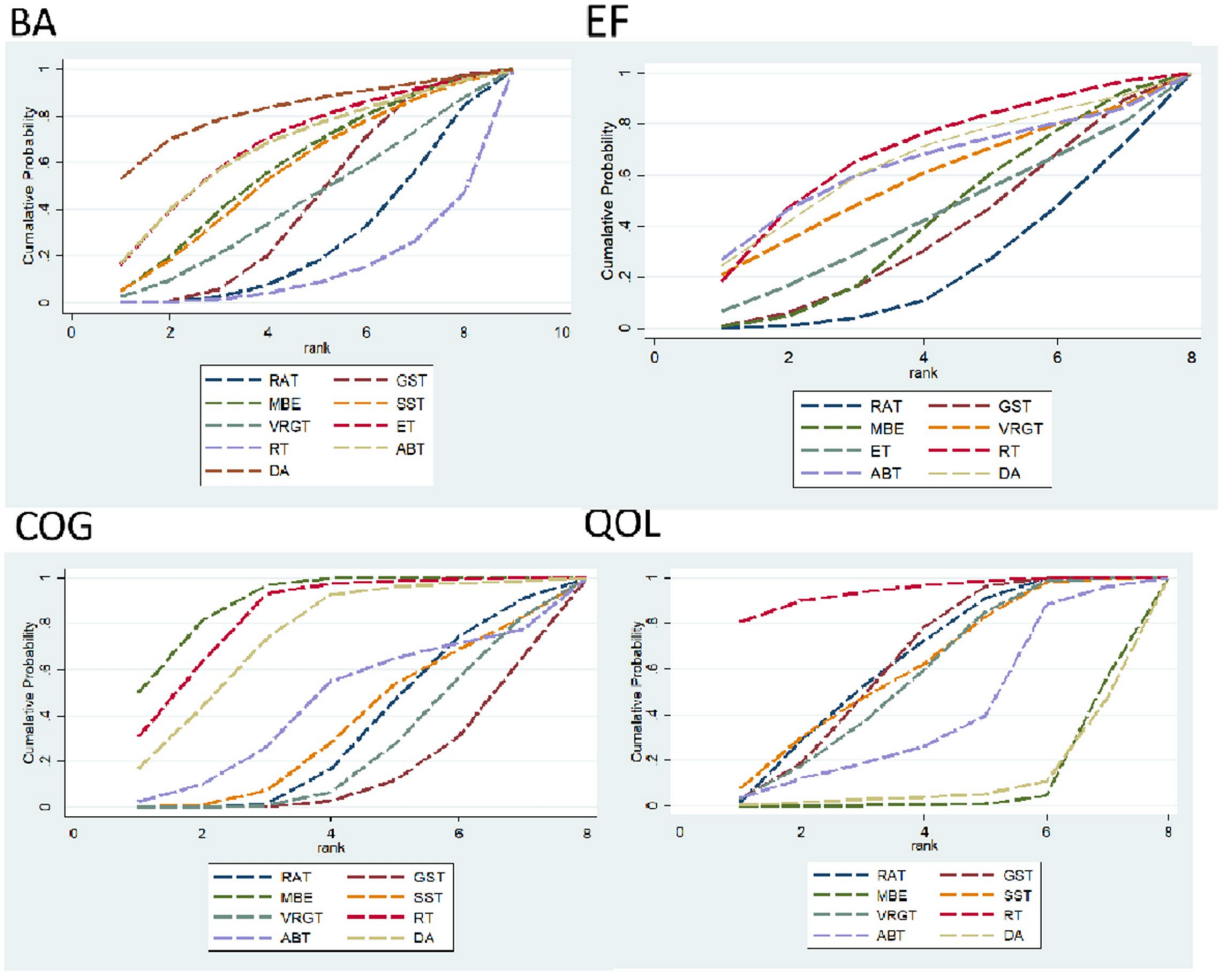
Area under the curve for cumulative ranking probability.

RAT ([SUCRA] = 25.4), GST ([SUCRA] = 41.6), MBE ([SUCRA] = 57.2), SST ([SUCRA] = 55.0), VRGT ([SUCRA] = 42.1), ET ([SUCRA] = 67.4), RT ([SUCRA] = 13.1), ABT ([SUCRA] = 66.1), DA ([SUCRA] = 82.1). See Figure 8 and Tables 3, 4.

**Table 3:** Ranking the probability of nine exercise therapy.

Treatment	Balance		Treatment	Emotional function		Treatment	Cognitive		Treatment	Quality of life	
	SUCRA (%)	Rank		SUCRA (%)	Rank		SUCRA (%)	Rank		SUCRA (%)	Rank
RAT	25.4	8	RAT	23.5	8	RAT	33.0	6	RAT	63.8	2
GST	41.6	7	GST	37.1	7	GST	15.9	8	GST	63.5	3
MBE	57.2	4	MBE	41.9	6	MBE	89.7	1	MBE	8,9	8
SST	55.0	5	VRGT	57.7	4	SST	34.7	5	SST	61.3	4
VRGT	42.1	6	ET	42.8	5	VRGT	25.1	7	VRGT	57.3	5
ET	67.4	2	RT	68.6	1	RT	83.3	2	RT	94.3	1
RT	13.1	9	ABT	63.4	3	ABT	44.1	4	ABT	40.7	6
ABT	66.1	3	DA	64.9	2	DA	74.3	3	DA	10.3	7
DA	82.1	1									

**Table 4:** Network meta-analysis matrix of outcome.

Balance								
DA	−0.77 (−3.79, 2.24)	−0.78 (−3.82, 2.26)	−1.08 (−3.86, 1.69)	−1.14 (−3.93, 1.65)	−1.47 (−4.31, 1.38)	−1.45 (−4.03, 1.13)	−1.80 (−4.31, 0.72)	−2.20 (−4.75, 0.35)
0.77 (−2.24, 3.79)	ET	−0.01 (−2.43, 2.42)	−0.31 (−2.42, 1.80)	−0.36 (−2.43, 1.71)	−0.69 (−2.82, 1.43)	−0.67 (−2.34, 0.99)	−1.02 (−2.84, 0.79)	−1.43 (−3.52, 0.67)
0.78 (−2.26, 3.82)	0.01 (−2.42, 2.43)	ABT	−0.30 (−2.46, 1.86)	−0.36 (−2.55, 1.83)	−0.69 (−2.92, 1.55)	−0.67 (−2.44, 1.10)	−1.02 (−3.02, 0.99)	−1.42 (−3.41, 0.56)
1.08 (−1.69, 3.86)	0.31 (−1.80, 2.42)	0.30 (−1.86, 2.46)	MBE	−0.06 (−1.88, 1.76)	−0.38 (−2.27, 1.50)	−0.37 (−1.70, 0.97)	−0.71 (−2.25, 0.82)	−1.12 (−2.68, 0.44)
1.14 (−1.65, 3.93)	0.36 (−1.71, 2.43)	0.36 (−1.83, 2.55)	0.06 (−1.76, 1.88)	SST	−0.33 (−2.15, 1.50)	−0.31 (−1.62, 1.00)	−0.66 (−2.03, 0.71)	−1.06 (−2.86, 0.73)
1.47 (−1.38, 4.31)	0.69 (−1.43, 2.82)	0.69 (−1.55, 2.92)	0.38 (−1.50, 2.27)	0.33 (−1.50, 2.15)	VRGT	0.02 (−1.36, 1.40)	−0.33 (−1.82, 1.17)	−0.73 (−2.59, 1.12)
1.45 (−1.13, 4.03)	0.67 (−0.99, 2.34)	0.67 (−1.10, 2.44)	0.37 (−0.97, 1.70)	0.31 (−1.00, 1.62)	−0.02 (−1.40, 1.36)	GST	−0.35 (−1.36, 0.66)	−0.75 (−2.06, 0.56)
1.80 (−0.72, 4.31)	1.02 (−0.79, 2.84)	1.02 (−0.99, 3.02)	0.71 (−0.82, 2.25)	0.66 (−0.71, 2.03)	0.33 (−1.17, 1.82)	0.35 (−0.66, 1.36)	RAT	−0.40 (−1.89, 1.08)
2.20 (−0.35, 4.75)	1.43 (−0.67, 3.52)	1.42 (−0.56, 3.41)	1.12 (−0.44, 2.68)	1.06 (−0.73, 2.86)	0.73 (−1.12, 2.59)	0.75 (−0.56, 2.06)	0.40 (−1.08, 1.89)	RT
Emotional function								
RT	0.16 (−3.16, 3.49)	0.04 (−2.35, 2.43)	0.47 (−3.37, 4.32)	1.04 (−2.48, 4.55)	1.02 (−1.28, 3.32)	1.18 (−1.91, 4.26)	1.51 (−1.07, 4.09)	
−0.16 (−3.49, 3.16)	DA	−0.12 (−4.22, 3.97)	0.31 (−3.60, 4.22)	0.88 (−2.70, 4.46)	0.85 (−1.55, 3.25)	1.01 (−2.15, 4.18)	1.35 (−1.33, 4.02)	
−0.04 (−2.43, 2.35)	0.12 (−3.97, 4.22)	ABT	0.43 (−4.09, 4.96)	1.00 (−3.25, 5.25)	0.98 (−2.34, 4.29)	1.14 (−2.77, 5.04)	1.47 (−2.05, 4.99)	
−0.47 (−4.32, 3.37)	−0.31 (−4.22, 3.60)	−0.43 (−4.96, 4.09)	VRGT	0.57 (−2.28, 3.41)	0.54 (−2.54, 3.63)	0.70 (−1.59, 3.00)	1.04 (−1.81, 3.88)	
−1.04 (−4.55, 2.48)	−0.88 (−4.46, 2.70)	−1.00 (−5.25, 3.25)	−0.57 (−3.41, 2.28)	ET	−0.02 (−2.68, 2.64)	0.14 (−1.54, 1.82)	0.47 (−1.91, 2.85)	
−1.02 (−3.32, 1.28)	−0.85 (−3.25, 1.55)	−0.98 (−4.29, 2.34)	−0.54 (−3.63, 2.54)	0.02 (−2.64, 2.68)	MBE	0.16 (−1.90, 2.22)	0.49 (−0.69, 1.67)	
−1.18 (−4.26, 1.91)	−1.01 (−4.18, 2.15)	−1.14 (−5.04, 2.77)	−0.70 (−3.00, 1.59)	−0.14 (−1.82, 1.54)	−0.16 (−2.22, 1.90)	GST	0.33 (−1.36, 2.02)	
−1.51 (−4.09, 1.07)	−1.35 (−4.02, 1.33)	−1.47 (−4.99, 2.05)	−1.04 (−3.88, 1.81)	−0.47 (−2.85, 1.91)	−0.49 (−1.67, 0.69)	−0.33 (−2.02, 1.36)	RAT	
Cognitive								
MBE	−0.19 (−1.38, 0.99)	−0.38 (−1.46, 0.69)	−0.99 (−2.51, 0.53)	−1.21 (−2.04, −0.39)	−1.25 (−1.79, −0.71)	−1.33 (−1.98, −0.69)	−1.42 (−2.01, −0.84)	
0.19 (−0.99, 1.38)	RT	−0.19 (−1.62, 1.24)	−0.80 (−1.75, 0.16)	−1.02 (−2.26, 0.21)	−1.06 (−2.15, 0.03)	−1.14 (−2.13, −0.15)	−1.23 (−2.32, −0.15)	
0.38 (−0.69, 1.46)	0.19 (−1.24, 1.62)	DA	−0.60 (−2.32, 1.12)	−0.83 (−2.00, 0.34)	−0.87 (−1.80, 0.06)	−0.95 (−1.98, 0.08)	−1.04 (−2.07, −0.01)	
0.99 (−0.53, 2.51)	0.80 (−0.16, 1.75)	0.60 (−1.12, 2.32)	ABT	−0.23 (−1.79, 1.34)	−0.26 (−1.71, 1.19)	−0.34 (−1.72, 1.03)	−0.44 (−1.88, 1.01)	
1.21 (0.39, 2.04)	1.02 (−0.21, 2.26)	0.83 (−0.34, 2.00)	0.23 (−1.34, 1.79)	SST	−0.04 (−0.75, 0.68)	−0.12 (−0.86, 0.62)	−0.21 (−0.84, 0.41)	
1.25 (0.71, 1.79)	1.06 (−0.03, 2.15)	0.87 (−0.06, 1.80)	0.26 (−1.19, 1.71)	0.04 (−0.68, 0.75)	RAT	−0.08 (−0.53, 0.37)	−0.17 (−0.62, 0.28)	
1.33 (0.69, 1.98)	1.14 (0.15, 2.13)	0.95 (−0.08, 1.98)	0.34 (−1.03, 1.72)	0.12 (−0.62, 0.86)	0.08 (−0.37, 0.53)	VRGT	−0.09 (−0.54, 0.35)	
1.42 (0.84, 2.01)	1.23 (0.15, 2.32)	1.04 (0.01, 2.07)	0.44 (−1.01, 1.88)	0.21 (−0.41, 0.84)	0.17 (−0.28, 0.62)	0.09 (−0.35, 0.54)	GST	
Quality of life								
RT	−1.83 (−4.07, 0.41)	−1.87 (−4.61, 0.87)	−1.92 (−5.02, 1.17)	−2.05 (−4.89, 0.80)	−2.98 (−6.70, 0.74)	−6.29 (−10.85, −1.73)	−6.15 (−8.99, −3.31)	
1.83 (−0.41, 4.07)	RAT	−0.04 (−1.61, 1.53)	−0.10 (−2.24, 2.04)	−0.22 (−1.97, 1.53)	−1.15 (−4.12, 1.82)	−4.46 (−8.99, 0.07)	−4.33 (−7.11, −1.54)	
1.87 (−0.87, 4.61)	0.04 (−1.53, 1.61)	GST	−0.05 (−1.88, 1.78)	−0.18 (−1.58, 1.23)	−1.11 (−3.63, 1.41)	−4.41 (−9.21, 0.38)	−4.28 (−7.48, −1.08)	
1.92 (−1.17, 5.02)	0.10 (−2.04, 2.24)	0.05 (−1.78, 1.88)	SST	−0.12 (−2.35, 2.11)	−1.05 (−4.17, 2.06)	−4.36 (−9.37, 0.64)	−4.23 (−7.74, −0.72)	
2.05 (−0.80, 4.89)	0.22 (−1.53, 1.97)	0.18 (−1.23, 1.58)	0.12 (−2.11, 2.35)	VRGT	−0.93 (−3.82, 1.96)	−4.24 (−9.09, 0.61)	−4.11 (−7.39, −0.82)	
2.98 (−0.74, 6.70)	1.15 (−1.82, 4.12)	1.11 (−1.41, 3.63)	1.05 (−2.06, 4.17)	0.93 (−1.96, 3.82)	ABT	−3.31 (−8.72, 2.10)	−3.17 (−7.25, 0.90)	
6.29 (1.73, 10.85)	4.46 (−0.07, 8.99)	4.41 (−0.38, 9.21)	4.36 (−0.64, 9.37)	4.24 (−0.61, 9.09)	3.31 (−2.10, 8.72)	DA	0.13 (−3.44, 3.71)	
6.15 (3.31, 8.99)	4.33 (1.54, 7.11)	4.28 (1.08, 7.48)	4.23 (0.72, 7.74)	4.11 (0.82, 7.39)	3.17 (−0.90, 7.25)	−0.13 (−3.71, 3.44)	MBE	

*EF indicator*: The effective ranking of 14 types of exercise therapy for alleviating Emotional Function in elderly Parkinson’s patients, based on SUCRA values from highest to lowest, is as follows: RT ([SUCRA] = 68.6), DA ([SUCRA] = 64.9), ABT ([SUCRA] = 63.4), VRGT ([SUCRA] = 57.7), ET ([SUCRA] = 42.8), MBE ([SUCRA] = 41.9), GST ([SUCRA] = 37.1), RAT ([SUCRA] = 23.5). See Figure 8 and Tables 3, 4.

*COG indicators*: The effective ranking of eight types of exercise therapy on cognitive function in elderly Parkinson’s disease patients, MBE ([SUCRA] = 89.7), RT ([SUCRA] = 83.3), DA ([SUCRA] = 74.3), ABT ([SUCRA] = 44.1), SST ([SUCRA] = 34.7), RAT ([SUCRA] = 33.0), VRGT ([SUCRA] = 25.1), and GST ([SUCRA] = 15.9). For details, see Figure 8 and Tables 3, 4.

*QOL indicators*: The effective ranking of eight types of exercise therapy on the quality of life of elderly Parkinson’s patients: RT ([SUCRA] = 94.3), RAT ([SUCRA] = 63.8), GST ([SUCRA] = 63.5), SST ([SUCRA] = 61.3), VRGT ([SUCRA] = 57.3), ABT ([SUCRA] = 40.7), DA ([SUCRA] = 10.3), MBE ([SUCRA] = 8.9), see Figure 8 and Tables 3, 4.

### 3.6 Ranking of intervention effects for outcome indicators

In terms of balance, the DA group (SUCRA = 82.1%, ranked 1st), ET group (SUCRA = 67.4%, ranked 2nd), and ABT group (SUCRA = 66.1%, ranked 3rd) all showed significant improvements compared to the control group (RAT). Among these, the improvement effect of DA was the most prominent (SMD = 0.2, 95% CI [4.93, 3.14], *p* = 0.02), followed by ET (SMD = 0.61, 95% CI [0.73, 0.92], *p* < 0.0001). The efficacy of the MBE group was moderate (SUCRA = 57.2%, ranked 4th), with no significant difference from the control group (SMD = 1.29, 95% CI [1.27, 1.54], *p* = 0.87). The RT group had the weakest efficacy (SUCRA = 13.1%, rank 9), with a significant but poor effect compared to the control group (SMD = −0.5, 95% CI [0.15, 0.66]). See Table 3 for details.

In terms of emotional function, The RT group (SUCRA = 68.6%, ranked 1st), DA group (SUCRA = 64.9%, ranked 2nd), and ABT group (SUCRA = 63.4%, ranked 3rd) all showed significant improvement compared to the control group (RAT). Among these, the RT group showed the most significant effect (SMD = 1.02, 95% CI [0.67, 1.38], *p* < 0.0001), followed by the DA group (SMD = 0.85, 95% CI [−1.55, 3.25], *p* = 0.06) and the ABT group (SMD = 0.98, 95% CI [−2.34, 4.29], *p* = 0.56). The VRGT group (SUCRA = 57.7%, ranked 4th) showed a moderate effect (SMD = 0.70, 95% CI [−1.59, 3.00], *p* = 0.55), while the ET group (SUCRA = 42.8%, ranked 5th) and MBE group (SUCRA = 41.9%, ranked 6th) showed weaker but still significant effects (ET: SMD = 0.47, 95% CI [−1.91, 2.85], *p* = 0.70; MBE: SMD = 0.49, 95% CI [−0.69, 1.67], *p* = 0.42). The GST group (SUCRA = 37.1%, ranked 7th) and RAT group (SUCRA = 23.5%, ranked 8th) showed the smallest improvement and no significant difference from the control group (GST: SMD = 0.33, 95% CI [−1.36, 2.02], p = 0.70; RAT: SMD = 0.22, 95% CI [−1.53, 1.97], *p* = 0.81). See Table 3 for details. For a sensitivity analysis of quality of life, please refer to Appendices 3, 4.

In terms of cognition, the MBE group (SUCRA = 89.7%, ranked 1st), RT group (SUCRA = 83.3%, ranked 2nd), and DA group (SUCRA = 74.3%, ranked 3rd) all showed significant improvement compared to the control group (RAT). Among these, MBE showed the most prominent improvement (SMD = 1.29, 95% CI [1.27, 1.54], *p* < 0.0001), followed by RT (SMD = −0.5, 95% CI [0.15, 0.66], *p* = 0.006). The efficacy of the DA group was significant (SMD = 1.5, 95% CI [1.5, 3.1], *p* = 0.02). The efficacy of the ABT group was moderate (SUCRA = 44.1%, ranked 4th) and significantly different from the control group (SMD = −0.78, 95% CI [0.02, 1.34], *p* = 0.04). The GST group had the weakest efficacy (SUCRA = 15.9%, rank 8), with no significant difference from the control group (SMD = −0.08, 95% CI [−0.86, 0.71]). See Table 3 for details.

In terms of quality of life, the RT group (SUCRA = 94.3%, ranked 1st), ART group (SUCRA = 63.8%, ranked 2nd), and GST group (SUCRA = 63.5%, ranked 3rd) all showed varying degrees of improvement compared to the control group (RAT). Among these, RT showed the most significant improvement (SMD = 10.81, 95% CI [9.47, 12.14], *p* < 0.0001), followed by ABT (SMD = 1.11, 95% CI [0.62, 1.61], *p* < 0.0001). The efficacy of the SST group was moderate (SUCRA = 61.3%, ranked 4th) and significantly different from the control group (SMD = 0.37, 95% CI [0.04, 0.71], *p* = 0.03). The efficacy of the VRGT group was weak (SUCRA = 57.3%, rank 5), with no significant difference from the control group (SMD = 0.26, 95% CI [−0.22, 0.74], *p* = 0.28). The DA group had the worst efficacy (SUCRA = 10.3%, ranked 8th), with no significant difference from the control group (SMD = 0.14, 95% CI [−0.59, 0.87]). See Table 3 for details.

### 3.7 Publication bias and small sample size tests

For studies included in the network META analysis, corrected comparison funnel plots were used for small-sample effect estimation and publication bias testing. The included studies were largely symmetrical (see Figure 8 for details). For details on the Egger test, please refer to Appendix 5.

## 4 Discussion

**Figure 9:**
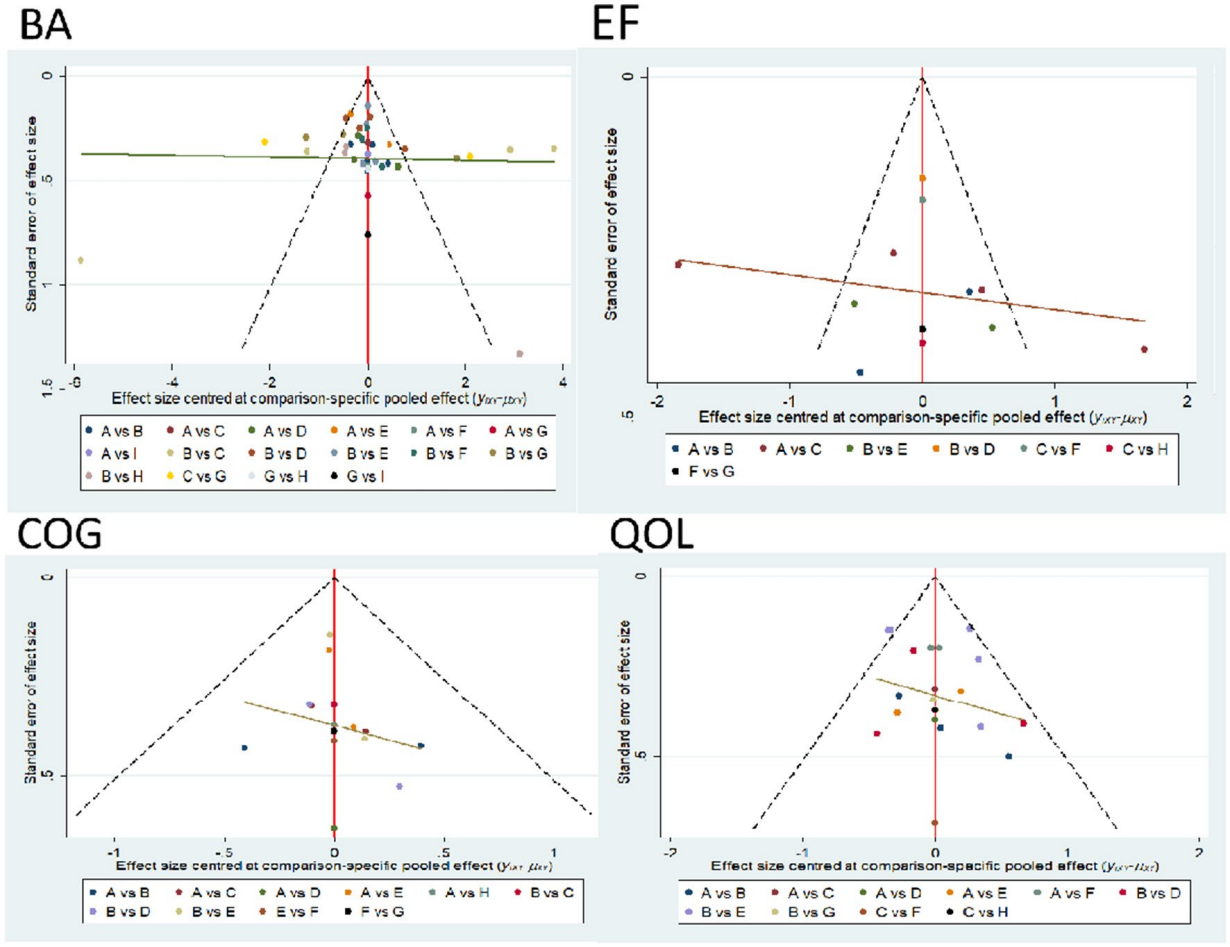
Comparison funnel plot for outcome indicators, A = RAT; B = GST; C = MBE; D = SST; E = VRGT; F = ET; G = RT; H = ABT.

In terms of balance, DA and ET are the most effective interventions for improving balance in elderly patients with Parkinson’s disease, with dance ranking highest (SUCRA = 82.1%) (SMD = −0.88, 95% CI [−1.52, −0.15]) (Figure 9). This finding is consistent with previous studies (24), as dance significantly improves balance function in Parkinson’s disease patients by combining rhythmic movements and cognitive tasks (25). DA outperforms traditional RAT and GST, possibly because it emphasizes both motor and cognitive engagement, meaning that interventions combining motor and cognitive elements can more effectively activate the basal ganglia-cortical loop (26). However, this study found that the efficacy of GST (SUCRA = 41.6%) was lower than expected, which may be due to the heterogeneity of the included studies (I^2^ = 74%) or the fact that some studies did not strictly standardize the intensity of gait training (27). In contrast, the efficacy of resistance training (RT) was limited (SUCRA = 13.1%), consistent with previous findings (28), which indicated that isolated strength training has limited effects on improving dynamic balance (SMD = 0.21, 95% CI [−0.05, 0.47]). These findings not only validate the advantages of DA and ET as multimodal interventions but also provide more precise evidence-based guidance for selecting balance rehabilitation programs in clinical practice (29).

In terms of emotional function, our research indicates that resistance training (RT) is the most effective intervention (SUCRA = 68.6%, SMD = 1.02, 95% CI [0.67, 1.38]), consistent with previous studies suggesting that RT reduces emotional disorders by regulating neurotrophic factors and enhancing psychological resilience (30). The significant benefits of dance (DA, SUCRA = 64.9%) and aquatic training (ABT, SUCRA = 63.4%) further support the role of multimodal exercise in emotional regulation, consistent with findings that rhythmic and aquatic activities alleviate anxiety and depression symptoms in Parkinson’s disease (PD) patients (31). However, the effects of mind–body exercise (MBE, SUCRA = 41.9%) were weaker than expected, contrasting with studies emphasizing the efficacy of MBE in reducing depressive symptoms through mindfulness and stress reduction (32). This discrepancy may stem from differences in intervention protocols, such as shorter training durations or insufficient emphasis on mindfulness components in the trials. Similarly, the limited impact of gait stability training (GST, SUCRA = 37.1%) and routine aerobic training (RAT, SUCRA = 23.5%) suggests that isolated movement-focused interventions may lack the cognitive-emotional engagement required to achieve significant emotional improvements. These findings highlight the superiority of RT and integrative approaches (e.g., DA, ABT) in PD emotional regulation, while emphasizing the need to develop standardized MBE protocols to maximize therapeutic potential (33). Future research should explore dose–response relationships and cultural adaptability to optimize emotional treatment outcomes.

In terms of cognitive function, our study shows that MBE is the most effective intervention (SUCRA = 89.7%) (SMD = −1.42, 95% CI [−2.01, −0.84]), consistent with previous studies. Mind–body exercises (such as tai chi and yoga) significantly improve executive function in Parkinson’s patients through dual-task training and neuroplasticity mechanisms (34). The cognitive improvement effects of RT (SUCRA = 83.3%) (SMD = −1.23, 95% CI [−2.32, −0.15]) are also consistent with previous research findings, which confirmed that resistance training enhances hippocampal function by upregulating BDNF (35). However, our study found that traditional aerobic training (RAT, SUCRA = 33.0%) had limited effects, which partially contradicts previous findings suggesting that long-term aerobic exercise has cognitive benefits for Parkinson’s patients (36). This discrepancy may stem from the lack of cognitive engagement components in the RAT interventions included in this study, suggesting that aerobic exercise alone may be insufficient to improve Parkinson’s-specific cognitive impairments (37). Overall, our results support the superiority of exercise-cognition integration interventions, aligning with current recommendations in Parkinson’s disease rehabilitation guidelines (38). However, the differences in effect sizes across specific exercise modalities require further validation through standardized intervention protocols.

In terms of quality of life, our study showed that RT was the most effective intervention (SUCRA = 94.3%, SMD = 1.83, 95% CI [0.41, 4.07]), which is highly consistent with previous research findings (39), confirming that resistance training significantly improves QOL by enhancing muscle strength and functional independence (40). The moderate effects of RAT (SUCRA = 63.8%) and GST (SUCRA = 63.5%) align with previous conclusions, supporting the baseline improvement effects of aerobic and gait training on quality of life (41, 42). However, we found that MBE (SUCRA = 8.9%) had a significantly lower effect size, inconsistent with previously reported data. This discrepancy may stem from the shorter duration of the MBE program in this study and the absence of home-based training components (43). Similarly, the low effect size of DA (SUCRA = 10.3%) contradicts previous studies, potentially reflecting differences in cultural adaptability and training intensity of dance interventions (44). These results emphasize that function-oriented resistance training may offer greater clinical advantages than traditional mind–body exercises or art therapy for improving quality of life in Parkinson’s disease, but treatment protocols should be optimized based on individual patient characteristics.

## 5 Limitations

This study has several limitations that should be acknowledged. The high heterogeneity observed in this study primarily stems from systemic differences across multiple levels: at the design level, the 55 RCTs exhibited significant variations in exercise type, intervention dosage, frequency, duration, and control group settings. For example, the design of dance and mind–body exercise programs may yield differing outcomes due to variations in cultural context or training standards. Additionally, the control groups included multiple cross-comparisons, leading to natural dispersion of effect sizes. In terms of patient characteristics, the study population ranged in age from 25 to 80 years, with disease duration varying from 1 to 13 years. Significant differences in exercise tolerance, disease stage, and cognitive reserve further amplified variability in intervention responses. However, this study ensured the robustness of the results by conducting a rigorous network meta-analysis (NMA) to integrate direct and indirect evidence, using a random-effects model to correct for heterogeneity, and providing relative effectiveness rankings of interventions via SUCRA rankings. Heterogeneity primarily reflects the true diversity of clinical practice and patient populations rather than flaws in the analytical methods. We assessed the risk of bias and data quality of these studies and found their impact on overall results to be limited. Second, due to limited literature, the lack of direct comparisons between certain interventions may weaken the robustness of network meta-analysis results. Third, restricting the analysis scope to Chinese and English literature may introduce language bias, potentially overlooking relevant studies in other languages, thereby limiting the generalizability of the findings. Fourth, The notably large standardized mean difference (SMD) observed in this study is uncommon in behavioral and clinical research, which may reflect outlier effects driven by specific sample characteristics or intervention dynamics (e.g., RT’s more direct targeting of quality-of-life factors compared to MBE). Generalizing these findings to other populations should be approached with caution until replication studies confirm the effect size. These factors highlight the need for future studies to adopt more standardized research protocols and expand the scope of literature inclusion. It is also necessary to explore whether RT can continue to bring about such significant improvements in quality of life in the long term compared to MBE.

## 6 Conclusion

This study demonstrates that exercise therapy can significantly improve limb balance, emotional function, cognitive function, and quality of life (QOL) in patients with Parkinson’s disease (PD). Among them, DA and ET have a significant effect on improving balance function., RT and DA perform optimally in regulating emotional function, MBE has the greatest advantage in enhancing cognitive function, and RT has the most significant effect on improving quality of life. These findings provide evidence-based guidance for rehabilitation practices in Parkinson’s disease, aiding in the development of individualized exercise programs tailored to different symptom domains and offering patients safe and effective non-pharmacological intervention options. By clarifying the relative efficacy of various exercise therapies, this study provides important reference for clinical decision-making, promoting the optimization of Parkinson’s disease management, thereby improving patient outcomes and reducing the disease burden. Future research should further optimize intervention protocols to maximize these benefits.

## Data Availability

The original contributions presented in the study are included in the article/Supplementary material, further inquiries can be directed to the corresponding author.
