# Supplementary material for: The effects of nine types of exercise rehabilitation therapies on improving limb balance, cognitive and emotional function, and quality of life in elderly patients with Parkinson’s disease: a network meta-analysis of 55 RCTs
Source: Front Neurol. 2025 Aug 26;16:1666552. doi: 10.3389/fneur.2025.1666552 (PMC12417168; doi:10.3389/fneur.2025.1666552)
Supplement: Supplementary file 1 [file Table_1.docx]

Appendix1

1.quality of life

inconsistency test

|  |
| --- |
| \| Coef. Std. Err. z P>\|z\| [95% Conf. Interval] |
|  |
| _y_B \| |
| _cons \| -.4045916 .2736523 -1.48 0.139 -.9409404 .1317571 |
|  |
| _y_C \| |
| _cons \| -.1593185 .3965374 -0.40 0.688 -.9365175 .6178806 |
|  |
| _y_D \| |
| des_BD \| -.6234487 .5913492 -1.05 0.292 -1.782472 .5355743 |
| _cons \| .0682051 .4682239 0.15 0.884 -.8494969 .9859071 |
|  |
| _y_E \| |
| des_BE \| -.9135678 .4313734 -2.12 0.034 -1.759044 -.0680915 |
| _cons \| .1402016 .2992354 0.47 0.639 -.446289 .7266922 |
|  |
| _y_F \| |
| des_CF \| 10.87221 .8556054 12.71 0.000 9.19525 12.54916 |
| _cons \| -.227436 .2213273 -1.03 0.304 -.6612296 .2063576 |
|  |
| _y_G \| |
| _cons \| -1.512508 .4098906 -3.69 0.000 -2.315879 -.709137 |
|  |
| _y_H \| |
| _cons \| -.3005461 .5954634 -0.50 0.614 -1.467633 .8665407 |

local inconsistency

| Side Direct Indirect Difference tau |
| --- |
| Coef. Std. Err. Coef. Std. Err. Coef. Std. Err. P>\|z\| |
| A B -.3278754 1.092762 .3354563 1.263846 -.6633317 1.670762 0.691 1.844986 |
| A C -.1585543 .4347618 -11.0239 .7886993 10.86535 .9005911 0.000 .3009889 |
| A D .0682051 1.899429 -.1885174 1.413113 .2567225 2.367428 0.914 1.856735 |
| A E .1138979 1.330261 -.5287794 1.280607 .6426774 1.846497 0.728 1.848469 |
| A F -.2274412 .2546539 10.64453 .8639871 -10.87198 .9007343 0.000 .3009874 |
| B D -.1077281 1.092374 .1496172 2.100356 -.2573453 2.36744 0.913 1.856736 |
| B E -.3067511 .8336508 .3384733 1.650065 -.6452244 1.848701 0.727 1.848634 |
| B G * -1.107592 1.287146 .0897545 44.80522 -1.197347 44.8237 0.979 1.784809 |
| C F 10.80539 .7467974 -.0689514 .503814 10.87434 .9008527 0.000 .3009902 |
| C H * -.1412496 1.823339 8.66302 63.38623 -8.804269 63.41237 0.890 1.784953 |

2.Cognitive

inconsistency test

|  |
| --- |
| \| Coef. Std. Err. z P>\|z\| [95% Conf. Interval] |
|  |
| _y_B \| |
| _cons \| -.1747454 .2296747 -0.76 0.447 -.6248994 .2754087 |
|  |
| _y_C \| |
| _cons \| 1.250004 .2736407 4.57 0.000 .7136782 1.78633 |
|  |
| _y_D \| |
| _cons \| .0361179 .363943 0.10 0.921 -.6771972 .7494329 |
|  |
| _y_E \| |
| _cons \| -.0817072 .2284543 -0.36 0.721 -.5294694 .366055 |
|  |
| _y_F \| |
| _cons \| 1.058162 .5555909 1.90 0.057 -.0307764 2.1471 |
|  |
| _y_G \| |
| _cons \| .2615185 .7386365 0.35 0.723 -1.186182 1.709219 |
|  |
| _y_H \| |
| _cons \| .8663758 .4739064 1.83 0.068 -.0624635 1.795215 |

local inconsistency

| Side Direct Indirect Difference tau |
| --- |
| Coef. Std. Err. Coef. Std. Err. Coef. Std. Err. P>\|z\| |
| A B .0770195 .3760851 -.3445826 .3102065 .4216021 .487396 0.387 .316984 |
| A C .8241882 .2492195 2.274717 .3640118 -1.450529 .4411519 0.001 6.84e-11 |
| A D .1140062 .7083401 .0089078 .4410874 .1050984 .8344481 0.900 .3173498 |
| A E .0664937 .2744318 -.3797419 .3934476 .4462356 .4779297 0.350 .2831655 |
| A H . . . . . . . . |
| B C 2.172376 .3225108 .7217417 .3010117 1.450635 .441159 0.001 5.71e-10 |
| B D .1943814 .3644394 .2995121 .7509152 -.1051307 .8344147 0.900 .317352 |
| B E -.0504603 .2686491 .3956581 .3934337 -.4461184 .4780183 0.351 .2831771 |
| E F * 1.140104 .5065868 .4056137 28.29156 .7344908 28.29609 0.979 .2929684 |
| F G * -.7965653 .4868274 -2.14421 63.24687 1.347645 63.24866 0.983 .2929634 |

3.Balance

inconsistency test

|  |
| --- |
| \| Coef. Std. Err. z P>\|z\| [95% Conf. Interval] |
|  |
| _y_B \| |
| _cons \| .2128839 .770865 0.28 0.782 -1.297984 1.723752 |
|  |
| _y_C \| |
| des_BC \| -.320821 2.078397 -0.15 0.877 -4.394405 3.752763 |
| _cons \| .4652326 1.714872 0.27 0.786 -2.895854 3.826319 |
|  |
| _y_D \| |
| des_BD \| .105029 1.524045 0.07 0.945 -2.882044 3.092102 |
| _cons \| .5201314 1.000007 0.52 0.603 -1.439846 2.480108 |
|  |
| _y_E \| |
| des_BE \| -.2845812 1.672074 -0.17 0.865 -3.561787 2.992624 |
| _cons \| .429656 1.209967 0.36 0.723 -1.941836 2.801148 |
|  |
| _y_F \| |
| des_BF \| -2.125568 2.15095 -0.99 0.323 -6.341353 2.090217 |
| _cons \| 2.525657 1.745037 1.45 0.148 -.8945526 5.945867 |
|  |
| _y_G \| |
| des_BG \| -1.066165 2.178102 -0.49 0.624 -5.335168 3.202837 |
| des_CG \| -1.794335 2.754253 -0.65 0.515 -7.192572 3.603903 |
| _cons \| .2118884 1.778928 0.12 0.905 -3.274746 3.698523 |
|  |
| _y_H \| |
| des_GH \| -3.698422 2.816554 -1.31 0.189 -9.218767 1.821923 |
| _cons \| 2.093185 1.313127 1.59 0.111 -.4804964 4.666865 |
|  |
| _y_I \| |
| des_GI \| 2.582059 3.096379 0.83 0.404 -3.486733 8.65085 |
| _cons \| .8838327 1.731039 0.51 0.610 -2.50894 4.276606 |

| Side Direct Indirect Difference tau |
| --- |
| Coef. Std. Err. Coef. Std. Err. Coef. Std. Err. P>\|z\| |
| A B .2169052 .7693211 .4616005 .7181346 -.2446953 1.052411 0.816 1.675669 |
| A C .4619328 1.705585 .7773007 .9011902 -.3153679 1.929032 0.870 1.675729 |
| A D .5200689 .9922036 .805849 1.023028 -.2857801 1.425147 0.841 1.676472 |
| A E .4295789 1.201143 .2579103 1.021822 .1716686 1.576976 0.913 1.677689 |
| A F 2.525657 1.694788 .3859562 1.101993 2.139701 2.021558 0.290 1.638394 |
| A G .2152456 1.765846 -.5491126 .8573698 .7643583 1.962981 0.697 1.670543 |
| A I .8838327 1.694932 3.055668 1.988887 -2.171835 2.613133 0.406 1.653324 |
| B C -.0614276 .8746991 1.074748 1.129993 -1.136175 1.429411 0.427 1.665941 |
| B D .4128044 .8479954 .1248079 1.146068 .2879965 1.425683 0.840 1.676499 |
| B E -.0675476 .8539542 .1024745 1.32598 -.1700221 1.577169 0.914 1.677696 |
| B F .1874981 .965415 2.321277 1.77517 -2.133779 2.020709 0.291 1.638367 |
| B G -1.067983 .9821068 -.458021 .943924 -.6099619 1.362191 0.654 1.669556 |
| B H 1.828474 .9634695 -3.120918 1.699673 4.949393 1.954169 0.011 1.505976 |
| C G -2.049342 1.192731 -.3650168 1.072893 -1.684326 1.604047 0.294 1.649754 |
| G H -1.818188 1.570602 3.136395 1.166029 -4.954583 1.956122 0.011 1.505994 |
| G I 3.256989 1.821123 1.0818 1.875128 2.175189 2.613917 0.405 1.653343 |

local inconsistency

4.Emotional Functions

local inconsistency

| Side Direct Indirect Difference tau |
| --- |
| Coef. Std. Err. Coef. Std. Err. Coef. Std. Err. P>\|z\| |
| A B * -.3352453 .8636033 .1625852 18.26999 -.4978304 18.29039 0.978 1.161089 |
| A C * -.4938614 .6018701 .6360614 18.27871 -1.129923 18.28862 0.951 1.161046 |
| B D * -.7025836 1.170406 .6692837 63.30135 -1.371867 63.31217 0.983 1.160904 |
| B E * -.1364547 .8578486 .6694654 44.77848 -.8059201 44.7867 0.986 1.160995 |
| C F * -1.019363 1.175181 .571944 28.31264 -1.591307 28.33701 0.955 1.161095 |
| C H * -.8545545 1.225039 .9564799 63.29036 -1.811034 63.30214 0.977 1.160851 |
| F G * .0372174 1.219061 3.025003 63.33142 -2.987786 63.34308 0.962 1.161028 |

Appendix 2


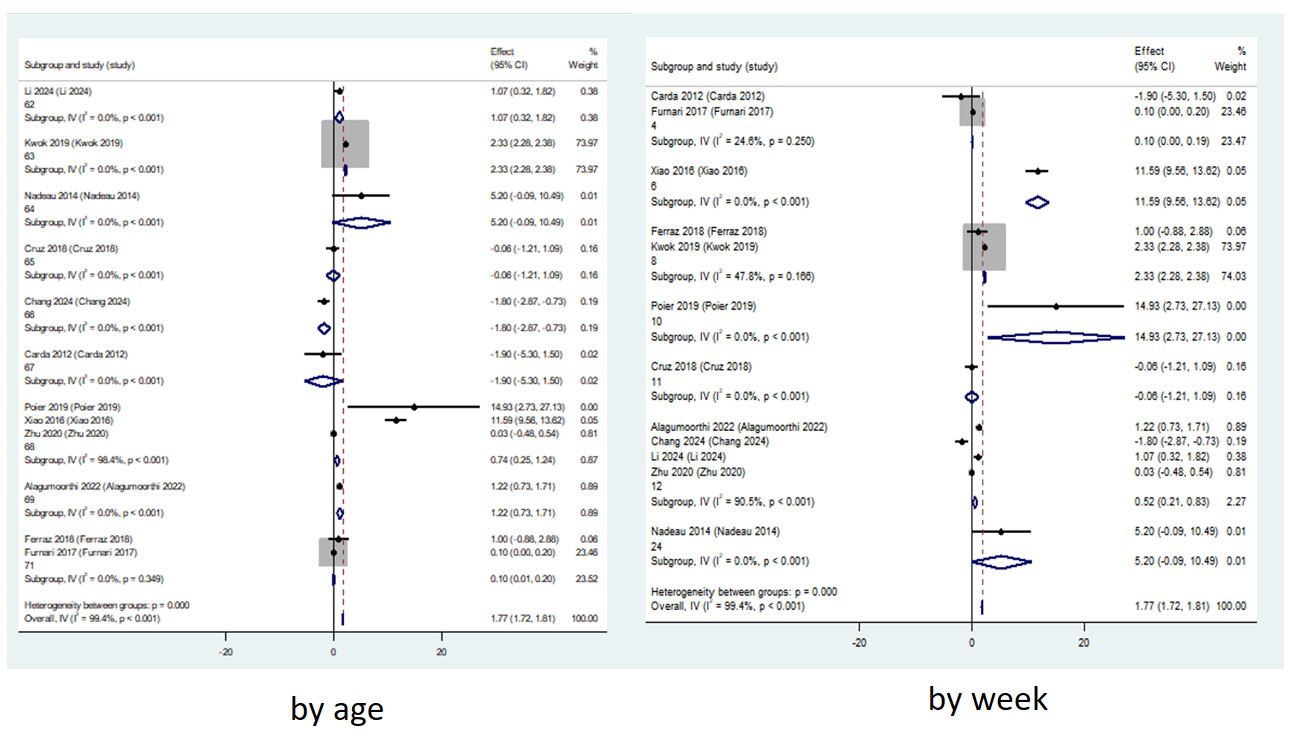


Subgroup analysis of Emotional Functions

| ------------------------------------------------------------------------------ |
| --- |
| _ES \| Coef. Std. Err. t P>\|t\| [95% Conf. Interval] |
| -------------+---------------------------------------------------------------- |
| age \| .437425 .137785 3.17 0.003 .157984 .7168659 |
| week \| -.0473381 .0975704 -0.49 0.630 -.24522 .1505438 |
| _cons \| -30.56562 9.287081 -3.29 0.002 -49.40069 -11.73054 |
| ------------------------------------------------------------------------------ |

meta regression of Emotional Functions


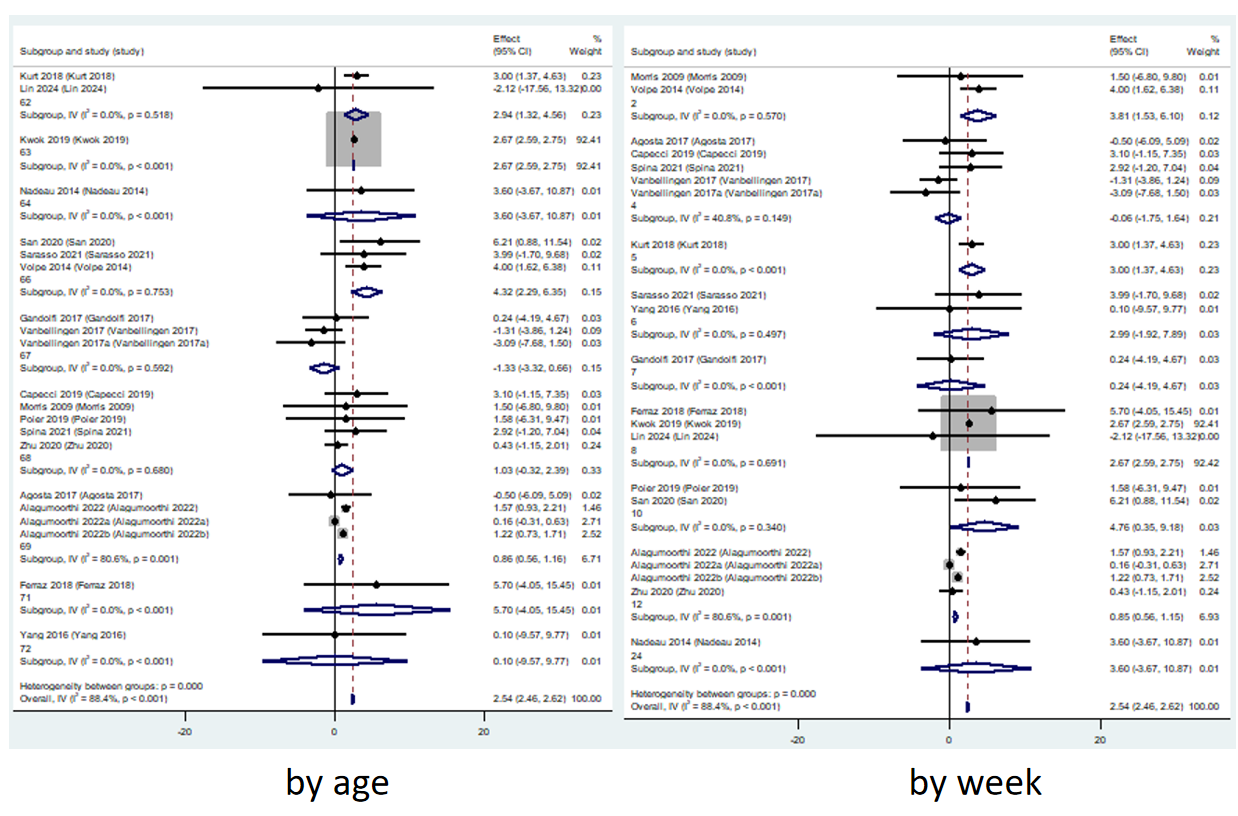


Subgroup analysis of quality of life

| ------------------------------------------------------------------------------ |
| --- |
| _ES \| Coef. Std. Err. t P>\|t\| [95% Conf. Interval] |
| -------------+---------------------------------------------------------------- |
| age \| -.3037508 .1342662 -2.26 0.036 -.5858337 -.0216679 |
| week \| .001792 .0993687 0.02 0.986 -.2069739 .210558 |
| _cons \| 21.84141 8.548366 2.56 0.020 3.881957 39.80086 |
| ------------------------------------------------------------------------------ |

meta regression of quality of life


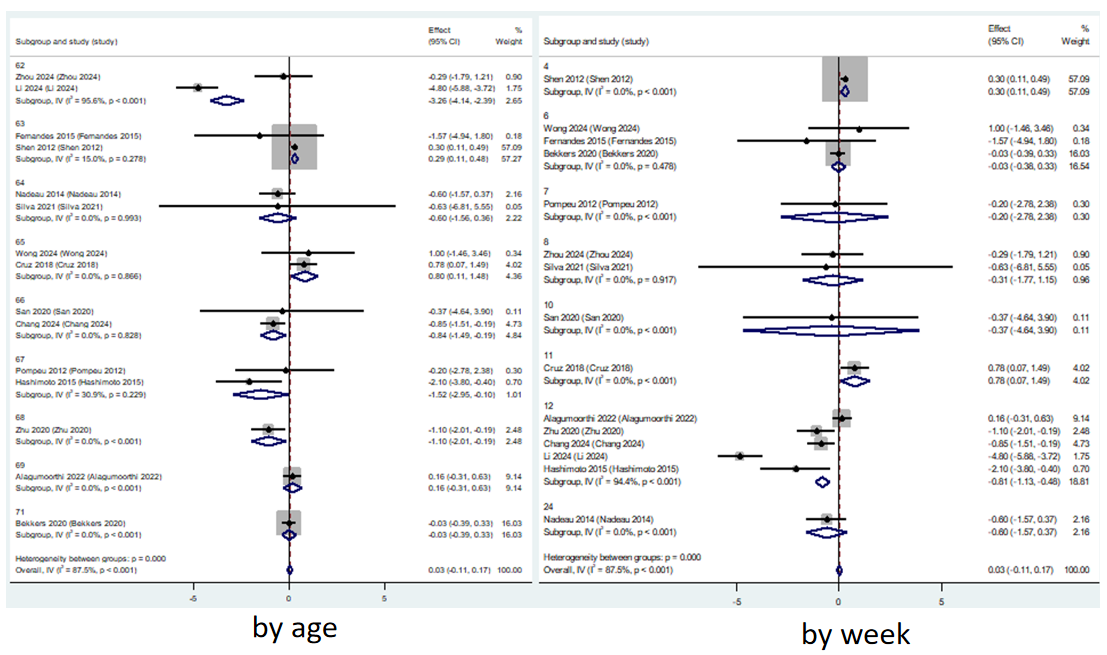


Subgroup analysis of cognitive

| ------------------------------------------------------------------------------ |
| --- |
| _ES \| Coef. Std. Err. t P>\|t\| [95% Conf. Interval] |
| -------------+---------------------------------------------------------------- |
| week \| -.0635246 .0862959 -0.74 0.476 -.2515472 .124498 |
| age \| .1500556 .155836 0.96 0.355 -.1894818 .489593 |
| _cons \| -9.875017 10.3442 -0.95 0.359 -32.4131 12.66307 |
| ------------------------------------------------------------------------------ |

meta regression of cognitive


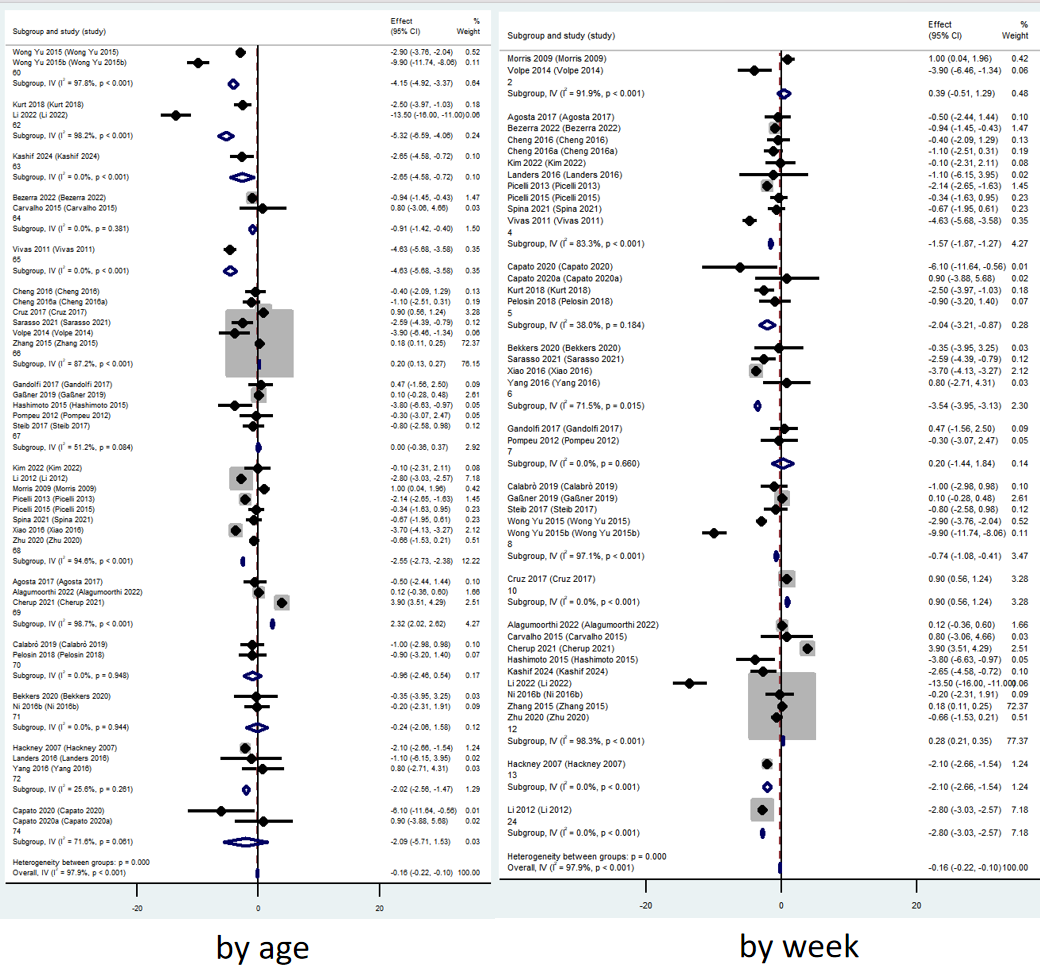


Subgroup analysis of balance

| ------------------------------------------------------------------------------ |
| --- |
| _ES \| Coef. Std. Err. t P>\|t\| [95% Conf. Interval] |
| -------------+---------------------------------------------------------------- |
| age \| .437425 .137785 3.17 0.003 .157984 .7168659 |
| week \| -.0473381 .0975704 -0.49 0.630 -.24522 .1505438 |
| _cons \| -30.56562 9.287081 -3.29 0.002 -49.40069 -11.73054 |
| ------------------------------------------------------------------------------ |

meta regression of balance

Appendix 3


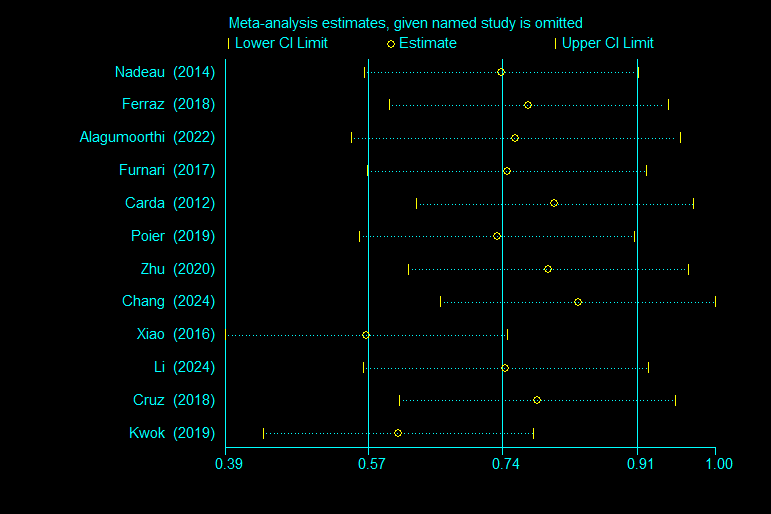


sensitivity analyses of Emotional Functions


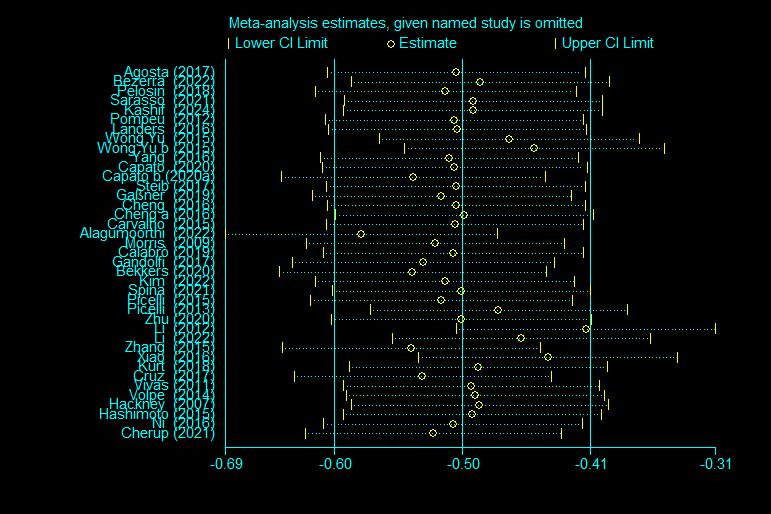


sensitivity analyses of balance


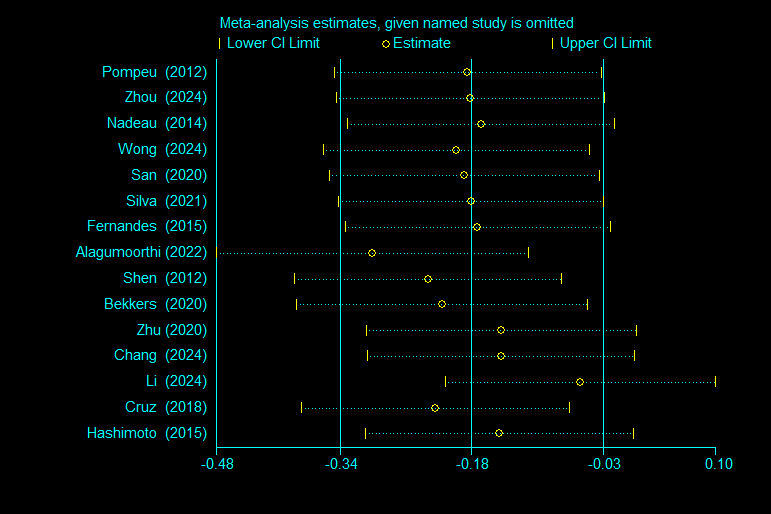


sensitivity analyses of cognitive


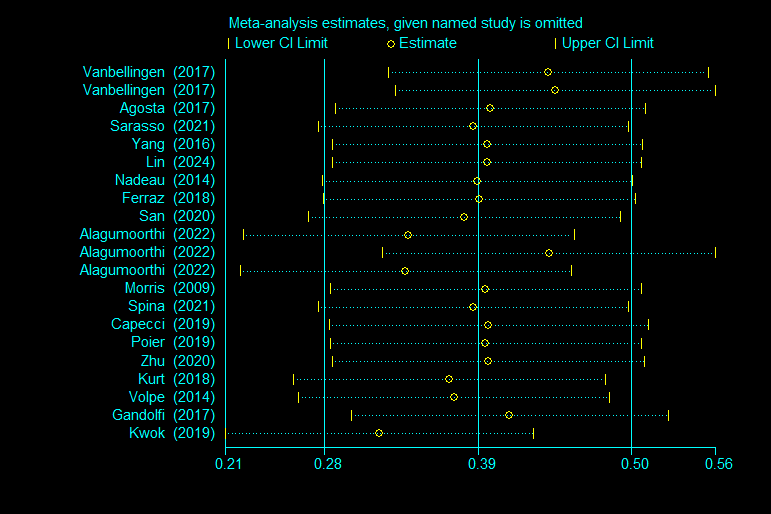


sensitivity analyses of quality of life

Appendix 4


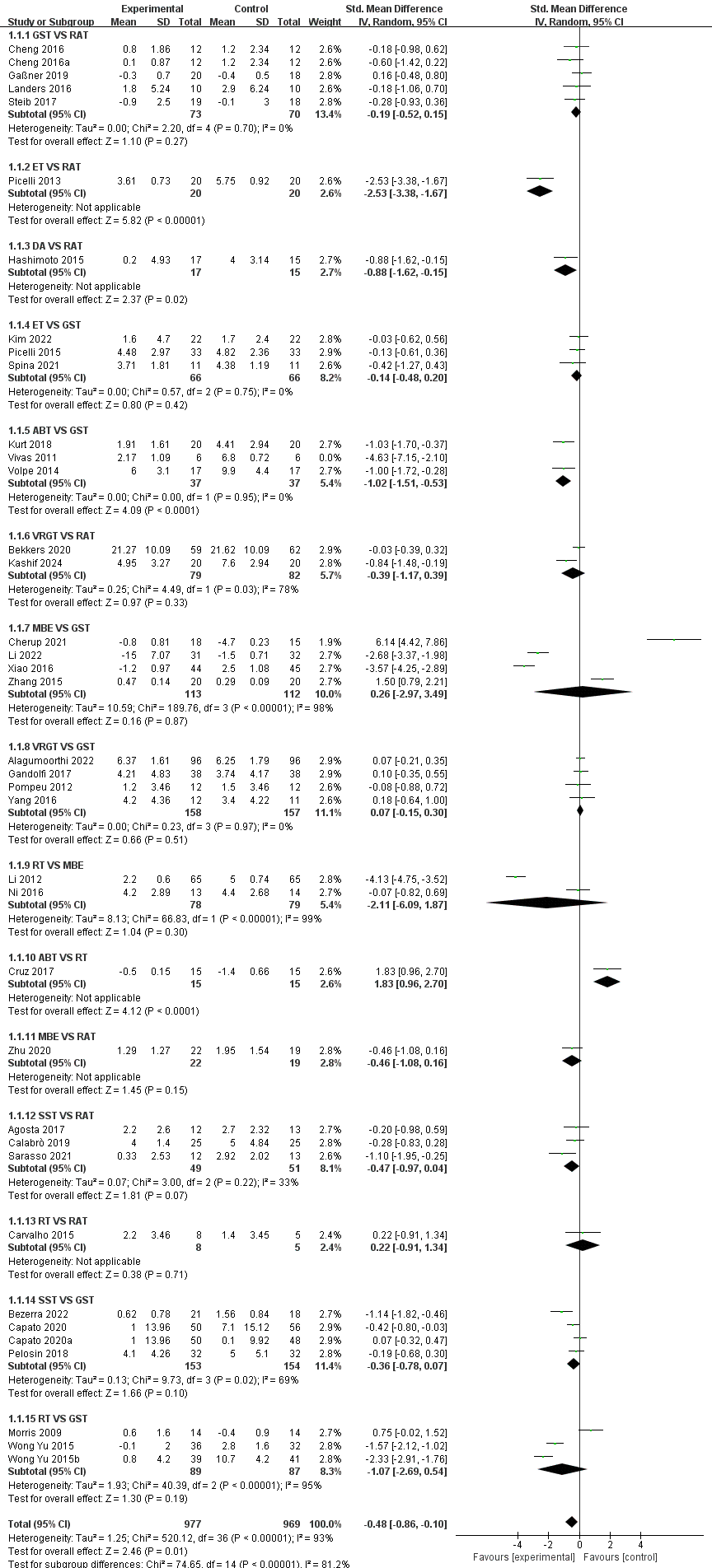


Forest of balance（exclusion Vivas 2011）

Appendix 5

|  |
| --- |
| Std_Eff \| Coefficient Std. err. t P>\|t\| [95% conf. interval] |
|  |
| slope \| 2.623019 .1190024 22.04 0.000 2.373945 2.872094 |
| bias \| -1.203123 .6553767 -1.84 0.082 -2.574842 .1685962 |

Egger test of quality of life

|  |
| --- |
| Std_Eff \| Coefficient Std. err. t P>\|t\| [95% conf. interval] |
|  |
| slope \| .3789273 .2638574 1.44 0.175 -.191102 .9489566 |
| bias \| -1.772424 .935676 -1.89 0.081 -3.793829 .2489813 |

Egger test of cognitive

|  |
| --- |
| Std_Eff \| Coefficient Std. err. t P>\|t\| [95% conf. interval] |
|  |
| slope \| .0943487 .2442701 0.39 0.702 -.4005895 .5892869 |
| bias \| -2.502224 1.235506 -2.03 0.050 -5.005597 .001148 |

Egger test of balance

|  |
| --- |
| Std_Eff \| Coefficient Std. err. t P>\|t\| [95% conf. interval] |
|  |
| slope \| 1.876023 .3597598 5.21 0.000 1.074428 2.677617 |
| bias \| -2.684012 4.406233 -0.61 0.556 -12.50171 7.133688 |

Egger test of Emotional Functions
